# Scalable machine learning approach to light induced order disorder phase transitions with ab initio accuracy

**DOI:** 10.1038/s41524-025-01614-5

**Published:** 2025-05-26

**Authors:** Andrea Corradini, Giovanni Marini, Matteo Calandra

**Affiliations:** Department of Physics, University of Trento, Povo, Italy

**Keywords:** Phase transitions and critical phenomena, Semiconductors, Structure of solids and liquids, Electronic structure

## Abstract

While machine learning excels in simulating material thermal properties, its application to order-disorder non-thermal phase transitions induced by visible light has been limited by challenges in accurately describing potential energy surfaces, forces, and vibrational properties in the presence of a photoexcited electron-hole plasma. Here, we present a novel approach that combines constrained density functional theory with machine learning, yielding highly reliable interatomic potentials capable of capturing electron-hole plasma effects on structural properties. Applied to photoexcited silicon, our potential accurately reproduces the phonon dispersion of the crystal phase and allows for molecular dynamics simulations of tens of thousands of atoms. We show that, at low enough temperatures, the non-thermal melting transition is driven by a soft phonon and the formation of a double-well potential, at odds with thermal melting being strictly first order. Our method paves the way to large-scale, long-time simulations of light-induced order-disorder phase transitions with ab initio accuracy.

## Introduction

Ultrafast pulses can induce order-disorder phase transitions that both challenge our comprehension of these phenomena and play a crucial role in technological applications. In the VO_2_ thermochromic compound, fs lasers can be used to promote a disordering of the vanadium dimers and a consequent insulator-metal transitions^1,2^. The laser-induced non-thermal melting and successive amorphization in phase change materials is accompanied by a large change in reflectivity, a property that is at the heart of the working principle of all-optical memories^3^. Along this line, a still poorly understood phenomenon is the possibility of tailoring the properties of liquid and amorphous systems by using non-thermal pathways. A prototypical example is photoexcited silicon, where the combination of ultrafast pumping and diffraction at a free-electron laser has led to the identification of a liquid-liquid phase transition occurring on the ps scale^4^.

Non-thermal melting of crystals is a ubiquitous and fascinating phenomenon arising in many semiconductors and insulators following intense ultrafast optical laser irradiation. When the ultrafast laser shines on the material, valence electrons are promoted to the conduction band, leaving a corresponding amount of holes in the valence band. With a timescale dictated by electron-electron and electron-phonon scatterings (usually tens or hundreds of femtoseconds^5^), a quasi-equilibrium thermalized electron-hole plasma is formed. As the fluences used in experiments generate a substantial occupation of the conduction-band anti-bonding states, the large forces exerted on the atoms lead to a structural destabilization of the lattice on a very fast scale, much faster than the electron-hole recombination time. As a consequence, non-thermal melting is easily distinguished from classical thermal melting as it happens in some hundreds of femtoseconds, as confirmed by ultrafast diffraction experiments at X-ray electron laser facilities^6^, and because it can occur at substantially lower lattice temperatures.

Despite the relevance of non-thermal melting for many applications, its understanding remains a challenge, even for the exemplary case of silicon. In this system, various experimental works report the appearance of non-thermal melting after promotion of 5–10% of valence electrons^7,8^, corresponding to an excited carrier density of ~10^22^/cm^3^. However, it is not yet clear if, similarly to what happens in the thermal case, non-thermal melting is a purely first-order phase transition or if it occurs via a soft-phonon mechanism. Theoretical simulations of non-thermal melting are a complex task due to the concomitant necessity of including out-of-equilibrium electrons and employing large simulation boxes.

Early works by Stampfli and Bennemann attempted to describe non-thermal melting in silicon by means of tight-binding models^9–11^ and by modeling the electron-hole plasma as a high-temperature Fermi-Dirac distribution. In ref. ^10^, the authors computed the transverse acoustic (TA) phonon frequency at the Brillouin zone (BZ) point L as a function of fluence, assuming a linear dispersion of the TA phonon over the whole BZ. They found that the TA frequency becomes imaginary around 8% photoexcited electrons/holes; they interpreted the non-thermal melting phenomenon as resulting from an instability of the elastic force constants (*c*_11_−*c*_12_) leading to a destabilization of the TA phonon over the whole BZ, including L.

A wide number of works then tried to describe the dynamics of non-thermal melting by means of first-principles molecular dynamics (MD), like the works of Silvestrelli et al.^12,13^ and Zijlstra et al.^14^, and classical MD with analytical potentials fitted on ab-initio forces^15–17^. However, similarly to refs. ^9–11^, in these works the electron-hole plasma has been mostly treated by considering an extremely large electronic temperature (in some cases exceeding 30,000 K^17^) and a single Fermi distribution for the electrons^12–17^. The same principle was followed by Plettenberg et al. when fitting an electronic temperature dependent neural network potential^18^. The description of the electron-hole plasma through a single Fermi distribution with extremely large electronic temperature, while straightforward and able to simulate the promotion of a fraction of electrons to excited quasiparticle states, is questionable for semimetals and semiconductors, where the electron-hole recombination rate is much slower than a phonon period^19^ and than the occurrence of non-thermal melting.

It was pointed out that a more sensible way to model the quasi-equilibrium photo-induced electron-hole plasma is to use two different Fermi distributions, one for the electrons and one for the holes, each one with its own Fermi level determined by charge conservation. Recently, and in agreement with Ref. ^19^, it has been shown that the use of a single Fermi distribution with a very large electronic temperature^20^ leads to incorrect structural properties of the photoexcited state and, most important, it leads to a systematic temperature-induced smoothening of the excited-state potential energy surface resulting in a suppression of all possible phonon softenings. This explains why the pioneering works of Silvestrelli et al.^12,13^ were able to reproduce the non-thermal melting phenomenon but (i) only keeping the volume of the simulation box fixed and (ii) without detecting any phonon softening. Another confirmation of the excessive smoothness of the potential energy surface under this approximation was given in Ref. ^17^, where short range analytical potentials were fitted by using force matching against ab initio simulations, but a high accuracy in the force constants was only obtained at very high electronic temperatures, where the potential energy surface is much smoother and thus easier to reproduce^17^. On the contrary, in Ref. ^20^, where a constrained density functional perturbation theory (cDFPT) scheme including two different Fermi levels and distributions for the photoinduced holes and electrons has been developed, it was shown that this approximation leads to structural and vibrational properties of the photoexcited state in excellent agreement with experiments in Te^20^, in GeTe^21^ and in SnSe^22^, where the prediction of a photoexcited phase transition was confirmed by successive experimental work^23^ with only a 1 mJ/cm^2^ error on the critical fluence.

In the case of photoexcited silicon, the inclusion of a quasi-equilibrium electron-hole plasma highlights a substantially more complicated description than the one suggested in Refs. ^9–11^, as the instability of the TA mode does not manifest itself as an instability of the (*c*_11_ − *c*_12_) force constant and does not occur at the *L* point, but at a momentum close to the zone center.

An alternative ab initio approach to simulate non-thermal melting is real-time time-dependent density functional theory (rtTDDFT) (see for example, Liu et al.^24^). This technique includes, in principle, the dynamics of the electrons without need to make any assumption on the model describing the quasi-equilibrium electron-hole plasma. However, this technique also presents some drawbacks: rtTDDFT equations have to be corrected by hand to impose Fermi’s golden rule and detailed balance on the electronic occupations. However, this violates energy conservation and requires another ad-hoc modification of velocities to reintroduce it^24^. Finally, simulations are restricted to small system sizes (64–216 atoms) due to their large computational cost. The results of rtTDDFT calculations are in agreement with experimental data for very small time scales (<100 fs from the laser pumping) but substantially depart from the measured Debye-Waller factors for larger times (>200 fs) (The agreement found in Fig. 2a of ref. ^24^ is fortuitous and it is related to the use of a small simulation box (64 atoms). The results of the 216-atom simulation box (that should be more representative of the thermodynamic limit), shown in Fig. S11, are substantially different and in disagreement with experiments for larger times, as also shown in Fig. 5 of the current work). Despite the discussed limitations, rtTDDFT simulations capture the general physics of carrier relaxation and show that 100 fs after pumping electrons and holes do indeed adopt two separate distributions (see Fig.1b in Ref. ^24^), in stark contrast with the large electronic temperature and single Fermi distribution modeling of the quasi-equilibrium electron-hole plasma.

In this work, we overcome these two shortcomings by developing a machine learning based molecular dynamics approach to simulate non-thermal melting. The method relies on two basic ingredients. The first is cDFPT as developed in Ref. ^19,20^, where the quasi-equilibrium photoexcited electron-hole plasma is treated with two Fermi distributions. The second is the use of machine learning potentials obtained by force matching with cDFPT forces in the presence of an electron-hole plasma. We demonstrate (i) a very high accuracy in our MD simulations at all fluences relevant for experiments and (ii) simulations of non-thermal melting on a ps time scale up to 32768-atom cells with limited computational cost, opening to a systematic screening of non-thermal melting across many families of semiconductors. On a small simulation box (64 atoms), our simulations are in excellent agreement with rtTDDFT calculations, meaning that the explicit treatment of electrons has small effects on the non-thermal melting process. However, our work demonstrates that substantially larger cells are needed to correctly simulate non-thermal melting at low ionic temperatures.

The novelty of our method is that, to the best of our knowledge, this is the first time where extremely accurate machine learning potentials accounting for the presence of a quasi-equilibrium electron hole plasma (two Fermi distributions with different Fermi levels, one for the holes and one for the electrons) are developed and employed. Previous works either attempted to simulate ultrafast phenomena by using a ground state setup^25^ or a Fermi distribution with an extremely large electronic temperature^17,18^.

Finally, we point out that our method is not limited to a particular class of machine learning potentials or to a particular semiconductor. All the developments are indeed easily transferrable to other systems undergoing non-thermal melting, such as phase change materials, VO_2_, or other semiconductors, provided that the density functional theory (DFT) exchange and correlation functional is accurate enough for these systems.

For what concerns the special case of silicon considered in this paper, we achieve a complete explanation of the mechanisms driving the onset of non-thermal melting. We develop a one-dimensional model capturing the essence of the transition. We show that non-thermal melting occurs in correspondence of the dynamical instability of the TA phonon of bulk crystalline silicon. Thus, the non-thermal melting transition is initiated by a soft phonon, in stark contrast with what happens in the thermal case, where the solid-liquid transition is strictly first order.

## Results

### Evaluation of the laser-induced electronic temperature

As many calculations^13–17^ assumed a very large electronic temperature, of the order of several eV, it is important to obtain an accurate estimate of the electronic temperature to be used in calculations for the case of a visible light source.

We consider a fs source having laser frequency of the order of *ℏΩ* = 2 eV (625 nm laser). The silicon optical bandgap is *Δ* = 1.2 eV. In previous works^26^, a qualitative assessment of the energy transferred by the laser to the electron-hole plasma was obtained from the effective excess energy of the electron hole-pairs, namely *E* = *ℏΩ* − *Δ* ≈ 0.8 eV. However, this argument does not account for the fluence (i.e., the energy transferred per unit surface). Namely, it assumes that regardless of what the fluence is, all the electrons receive an excess energy of 0.8 eV. In order to visualize why this is a large overestimation of the electronic temperature, one could consider the limiting case of a single photon shining on a cm^2^ size sample. Clearly the increase in temperature for the entire electronic system would be almost zero. However, the previous argument still predicts an electronic temperature of 0.8 eV for the electron-hole plasma.

A more reliable (over)estimation of the electronic temperature can be obtained by assuming that all the laser energy is transferred to the electronic system in a quasiparticle picture, namely

1 $$\begin{array}{ll}E=(\hslash \Omega -\Delta )\times {N}_{e}\\ \quad=\mathop{\int}\nolimits_{-\infty }^{\Delta /2}\epsilon g(\epsilon ){f}_{v}(\epsilon )d\epsilon +\mathop{\int}\nolimits_{\Delta /2}^{\infty }\epsilon g(\epsilon ){f}_{c}(\epsilon )d\epsilon \\ \qquad-\mathop{\int}\nolimits_{-\infty }^{+\infty }\epsilon {g}_{0}(\epsilon )f(\epsilon )d\epsilon, \end{array}$$

where *N*_*e*_ ≈ 0.05–0.4 is the number of excited electrons per atom, *f*_*c*_(*ϵ*) and *f*_*v*_(*ϵ*) are the photoexcited electrons (*c* = conduction) and hole (*v* = valence) Fermi functions, respectively, and *g*(*ϵ*) is the density of states of the photoexcited system. The quantities *g*_0_(*ϵ*) and *f*(*ϵ*) are the ground state density of states and Fermi function, respectively. As experiments are carried out at room temperature, *f*(*ϵ*) can be well approximated by a step function. Moreover, we can neglect the band deformation induced by the laser, and we can assume *g*_0_(*ϵ*) = *g*(*ϵ*). Assuming the same temperature *T*_*e*_ for holes and electrons and replacing in *g*(*ϵ*), *g*_0_(*ϵ*), *f*_*v*_(*ϵ*), *f*_*c*_(*ϵ*) the corresponding quantities calculated from first principles, we can invert the relation and obtain *T*_*e*_ as a function of the incoming laser energy, the gap and the photocarrier concentration. We obtain that for *T*_*e*_ = 0.01 Ry ~ 1579 K the estimated excess energy is of the order of 1.37 eV, in good agreement with typical laser frequencies that are used in photoexcitation experiments. For this reason, we adopt an electronic temperature of 0.01 Ry in the continuation of our work.

### Silicon in the absence of photoexcitation

We fit a gaussian approximation potential (GAP)^27^ to accurately learn the potential energy surface of ground state (GS), i.e. non photoexcited silicon. The GAP potential is trained on a dataset comprising bulk crystalline, liquid and amorphous structures. Details about dataset generation and machine learning potential fitting are given in the Section “Methods”.

**Fig. 1:**
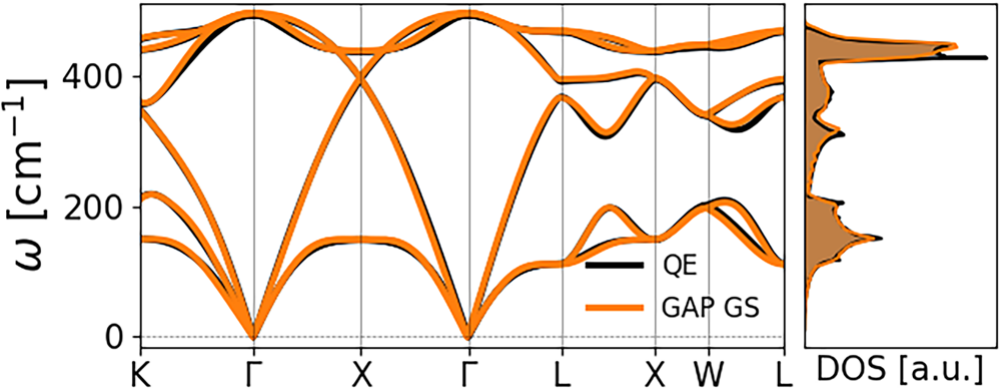
Phonon dispersion and density of states (DOS) of bulk Si in the diamond structure in the absence of photoexcitation. Black lines refer to a QE linear response calculation on a 12 × 12 × 12 phonon momentum grid. Orange lines refer to the finite displacement phonons computed on a 8 × 8 × 8 supercell (1024 atoms) by using our GAP potential.

In order to validate the GAP potential for the solid phase, we compare the bulk Si (diamond structure) calculation of the harmonic phonon dispersion obtained with the GAP GS potential and the QE DFPT calculation. The comparison is shown in Fig. 1, demonstrating the accuracy of our setup for the crystalline phase.

**Fig. 2:**
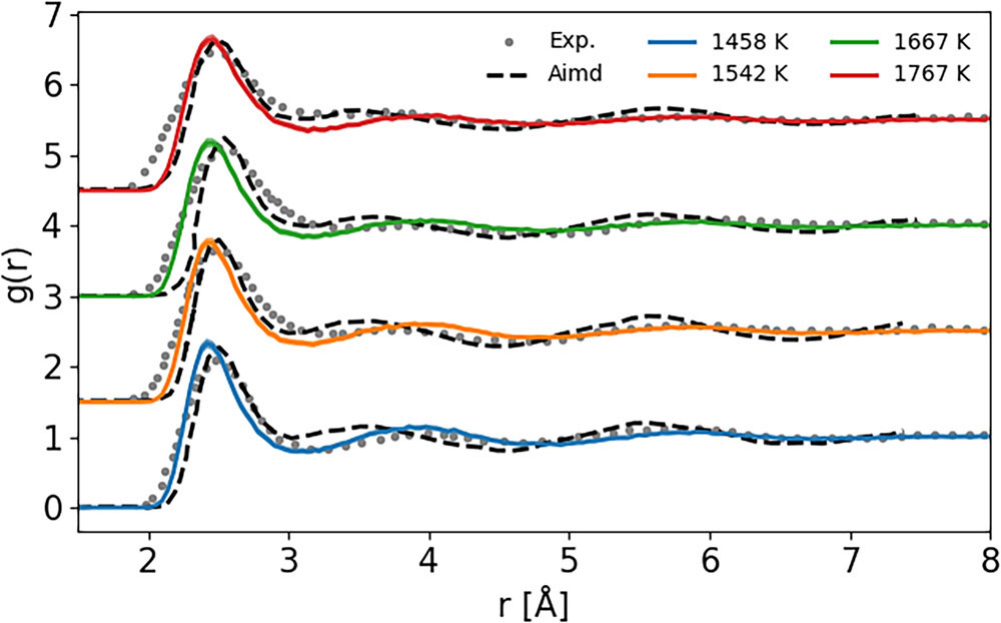
Equilibrium RDF from MD simulations in the constant temperature-constant pressure (NPT) ensemble using our developed GAP potential at zero pressure and various temperatures. Results are compared with experimental data (gray points) and AIMD simulations (dashed black lines) from ref. ^48^. The curves for *T* = 1542, 1667, and 1767 K are shifted upwards by 1.5, 3, and 4.5, respectively.

In order to validate our potential for the liquid phase, we run some MD simulations of liquid silicon and compare in Fig. 2 the resulting radial distribution functions (RDF) with available experimental data and ab initio molecular dynamics (AIMD). Our potential leads to an RDF in good agreement with both experimental data and AIMD results. The overall accuracy of the GAP GS potential is comparable to AIMD, taking into account the different approximations for the exchange correlation used, as discussed in Supplementary Section 2B.

In order to validate the capability of the potential to describe thermal melting, we run an MD simulation on a solid-liquid interface to estimate the melting point of ground-state silicon at zero pressure. Our interface simulation locates it around 1520–1530 K, that is approximately 150 K lower than the experimental one^28^. The confidence interval given here must not be interpreted in a strict statistical sense. The melting temperature estimation is only a sanity check of our GAP potential, and the purpose is not to evaluate the transition temperature with great accuracy, performing an accurate size scaling. Nevertheless, our result for the melting temperature is in very good agreement with the estimate given in Ref. ^29^ and with ab initio molecular dynamics using the generalized gradient approximation to the exchange and correlation functional, which estimates the melting temperature as 1492 ± 50 K^30^. Finally, our results represent a relevant improvement compared to previous simulations with analytical potentials in Ref. ^17^, which estimate the melting temperature at zero pressure as 1199 ± 2 K^17^. We conclude that the GAP potential for silicon accurately describes both the crystalline and liquid phases of silicon, and it also succeeds in predicting the melting temperature.

### Photoexcited silicon phases

Our goal is to model the time evolution of silicon samples after irradiation by a femtosecond laser with frequency *ℏΩ* ~2 eV for various fluences. We consider 7 different laser fluences up to a maximum value of 56.5 mJ/cm^2^, as usually chosen in experiments^7,8,31^. This implies fitting one GAP potential for each fluence, which will be used to reproduce the time evolution of silicon samples after irradiation by a laser with such fluence. For ease of reading, each GAP potential will be referred to with the corresponding value of laser fluence from now on. Dataset generation must then take into account the impact of the different laser fluences on the electronic distribution(s) in the crystal, liquid, and amorphous phases, treating the gapped diamond phase and gapless disordered phases differently.

Regarding the diamond phase, photoexcitation directly induces the formation of a high-density quasi-equilibrium thermalized electron-hole plasma, so it must be regarded as a photoexcited insulator. As such, electrons have slow recombination rates and calculations of total energies and forces are consequently performed in the two Fermi level approach of Ref. ^20^ with the same electronic temperature of 0.01 Ry ≈ 1579 K for electrons and holes. As estimated in subsection “Evaluation of the laser-induced electronic temperature” of section “Results” this value corresponds quite well with the excess energy of an electron-hole plasma after photoexcitation by a 2 eV optical laser. As reported by Harb et al.^8^ (see e.g. Fig. 4 in their work), the relation between femtosecond laser fluence and the amount of electrons promoted to the conduction band in the diamond phase can be approximated as linear, with proportionality constant 141.25 mJ/cm^2^ per valence electron per Si atom. As each silicon atom has 4 valence electrons, increasing the fluence from 0 to 56.5 mJ/cm^2^ is equivalent to promoting 0–0.4 electrons (0–10% of the electrons) per Si atom to the conduction band and to injecting a similar amount of holes in the valence band. The relation between laser fluence and photoexcited electrons in the diamond phase is reported in Table 1.

**Table 1:** Absorbed pump fluence and corresponding number of photoexcited electrons $N_e$ per Si atom in the gapped diamond phase

*N*_*e*_/Si	0.05	0.1	0.15	0.2	0.25	0.3	0.4
*N*_*e*_/Si [%]	1.25	2.5	3.75	5	6.25	7.5	10
Fluence [mJ/cm^2^]	7.1	14.1	21.2	28.2	35.3	42.3	56.5

We assume a linear dependence between them and use a proportionality coefficient of 141.25 mJ/cm^2^ per valence electron per Si atom, as done in ref. ^8^. The damage threshold is approximately 6 mJ/cm^2 ^^8^.

On the contrary, in our framework, the amorphous (semimetallic) and liquid (metallic) phases are obtained after disordering of the diamond phase due to the photoexciting laser. In silicon, disordering also implies bandgap closure, which favors electronic recombination on a fast timescale. As such, amorphous and liquid phases can be considered to be well described by a single Fermi distribution with a single Fermi level, as it is typically the case in metals^19,32^. For this reason, we calculate total energies and forces in these phases by adopting a single Fermi distribution, and we scale the electronic temperature linearly with the fraction *x* of promoted electrons, as shown in Table 3. This is done to retain coherence between gapped and non-gapped configurations in database generation, assuming that a higher fraction of photoexcited electrons in the conduction band of diamond silicon would result in a higher electronic temperature after recombination in the disordered phase.

It is important to point out, however, that while the choice of the correct thermal distribution of the photoexcited carriers is crucial in the crystalline phases, its effect is much less important in the liquid and amorphous phases, as disorder tends anyway to smear out the potential energy surface.

With the described databases we then fit GAP potentials for silicon in presence of photoexcitation. Parity plots for energy, forces and stress are illustrated in Supplementary Fig. S3 for the case of 0.2 photoexcited electrons/holes per Si atom.

**Fig. 3:**
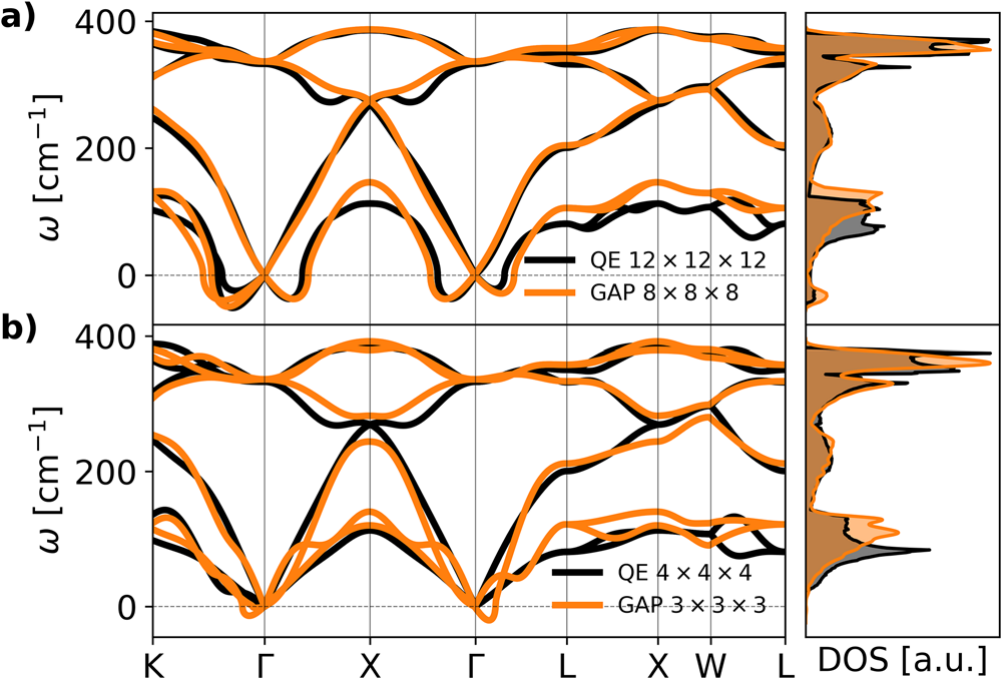
Phonon dispersion and density of states (DOS) for bulk crystalline silicon at a photoexcitation corresponding to 0.2 electrons/holes per Si atom. Black lines refer to QE cDFPT calculations and orange lines to finite displacement phonons computed with the GAP potential. **a** well converged phonon dispersion on a 12 × 12 × 12 cDFPT phonon momentum grid and 8 × 8 × 8 finite displacement supercell; **b** phonon dispersion out of convergence on a coarser (4 × 4 × 4) phonon momentum grid and smaller (3 × 3 × 3) supercell.

**Fig. 4:**
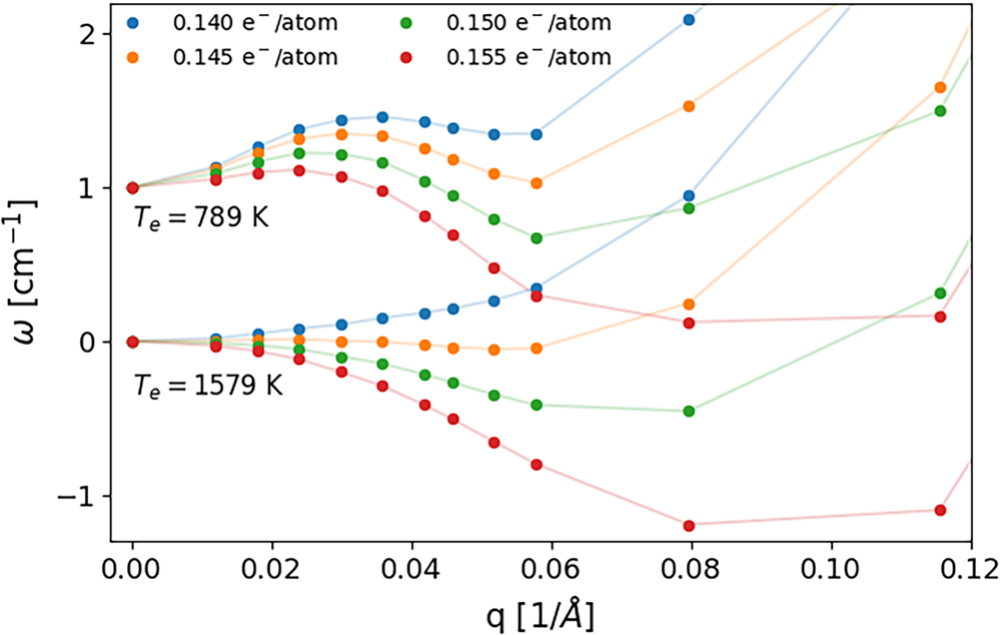
TA phonon dispersion in the vicinity of *Γ* along the *Γ*L direction. Phonons are plotted for 4 different fluences (expressed by the fraction of excited carriers per atom) and for two different electronic temperatures. Data at *T*_*e*_ ≈ 789 K are upshifted by 1 cm^−1^ for ease of reading. At low electronic temperature, it is clear that the phonon instability is localized and the sound velocity is still positive. Points refer to exact cDFPT calculations, while lines are just a guide to the eye. As a reference, BZ point L is defined by *q* = 1.00 Å^−1^.

The accuracy of the potential is demonstrated in Fig. 3a, where we perform a finite difference phonon calculation^33^ over an 8 × 8 × 8 supercell (1024 atoms) and we compare it with a linear response harmonic calculation on a 12 × 12 × 12 q-point grid at a fluence of 28.2 mJ/cm^2^ (corresponding to 0.2 photoexcited electrons/holes per Si atom) in the presence of an electron-hole plasma modeled by two Fermi distributions, one for the holes and one for the electrons. Both calculations provide well-converged results. As it can be seen in Fig. 3a, the agreement is excellent, and both calculations show the emergence of a structural instability via a soft TA phonon in the harmonic potential. The TA instability is due to a Kohn anomaly induced by a Fermi surface nesting, occurring very close to the zone center due to the low number of photocarriers in the valence and conduction bands and the consequent small Fermi vectors, but with a finite Fermi velocity. A more refined analysis of the Kohn anomaly with denser *q* sampling is performed in Fig. 4, where we plot the TA branch frequencies in the *Γ*L direction as a function of fluence with the same electronic temperature of 0.01 Ry = 1579 K used in dataset generation, showing the appearance of a Kohn anomaly that deepens as the fluence increases. However, the relatively high electronic temperature smears out the anomaly until reaching *Γ*, so that the sound velocity becomes negative as the anomaly appears. The figure also shows the same phonon dispersion recomputed with a lower electronic temperature (0.005 Ry = 790 K) and a suitably denser k-point grid. This calculation highlights the locality in reciprocal space of the anomaly and clearly shows that the sound velocity is always positive, even for the highest fluence reported. This result accurately describes the Fermi surface nesting origin of the TA phonon softening as fluence is increased and points to a second-order phase transition at the minimal critical fluence. At larger fluences, far above the critical one, or at extremely large electronic temperatures, the situation could be different, with the TA mode going completely imaginary in the full BZ. More details about the dependence of the phonon dispersion on the electronic temperature and on the treatment of the electron-hole plasma are reported in Supplementary Section S4.

We underline once more that this effect cannot be captured using a large electronic temperature of the order of tens of thousands of K, as done in previous works, since the potential energy surface is smeared out. This is confirmed by the fact that in Fig. 4 an electronic temperature of 0.01 Ry = 1579 K is already starting to hide the true Kohn anomaly origin of the softening. In fact, previous works in the literature only discussed the overall softening of the TA phonon over the BZ. For example, Recoules et al.^34^ showed that the TA branch in the *Γ*L and *Γ*X direction is unstable everywhere by modeling the electron-hole plasma as a hot Fermi distribution with a temperature of 25,000 K. Stampfli and Bennemann modeled the electron-hole plasma similarly in a tight-binding fashion; however, they assumed a linear dispersion of the TA phonon and found its softening at 8% photoexcited electrons/holes, i.e., 0.32 electrons/holes per atom. Fig. 4 shows that the softening appears at much lower fluences (3.5–3.9% photoexcited electrons/holes) at intermediate *q* vectors.

Finally, in Fig. 3b we also plot, as a reference, the phonon dispersion extracted from the GAP potential and in linear response for an excitation corresponding to 0.2 electrons/holes per Si atom, but on a 3 × 3 × 3 simulation box (54 atoms), and on a 4 × 4 × 4 phonon momentum grid in the case of linear response. These small cell/small phonon momentum grid calculations completely fail in detecting the relevant phonon instability of the low-energy mode. This demonstrates that small supercells are unable to correctly capture lattice dynamics during non-thermal melting and illustrates the need to use larger cells.

Overall, our potential very accurately captures the structural and vibrational properties of Si in the presence of photoexcitation.

### Molecular dynamics simulations of non-thermal melting

We investigate non-thermal melting in silicon by performing several MD simulations. We consider simulation boxes up to 32768 atoms. We set the initial condition for every MD simulation with all atoms in the ideal lattice sites of bulk diamond silicon. Atomic velocities are randomly distributed according to the Maxwell-Boltzmann distribution at a lattice temperature *T*_*l*_(0), i.e., the lattice temperature *before* photoexcitation. As we are interested in describing non-thermal melting in the irradiated area of a sample, before heat and mechanical work can start to propagate to other non-irradiated regions of the sample, we run MD in the microcanonical ensemble. In this way, we require that the system is fully decoupled from its surroundings after laser irradiation. The conserved energy in our MD simulations is the sum of the nuclear kinetic energy *K*_*I*_ and the electronic Mermin free energy *F*_el_ = *E* − *TS*, where *E* is the internal energy and *T* and *S* are respectively the temperature and entropy of the electron and hole Fermi distributions. For this reason, we will label the microcanonical ensemble as NV(*K*_*I*_ + *F*_el_). This comes from the fact that our GAP potentials have been trained on Mermin free energies and ionic forces computed by application of the Hellmann-Feynman theorem on the internal energy *E*, as routinely implemented in ab initio codes. We point out that these forces represent the correct gradient of the Mermin free energy in the presence of a finite electronic temperature, as demonstrated by Wentzcovitch et al.^35^. To the best of our knowledge, all previous ab initio calculations used to study non-thermal melting defined energies and forces in the same way, i.e., from the Mermin functional. In Supplementary Section S2A we show more details about the convenience of this choice. In particular, we show that the error introduced by the electronic entropy term in the Mermin free energy at the temperatures considered in this work is smaller than the accuracy of the GAP potentials on forces and energies, meaning that at all effects the dynamics is compatible with the one that would be obtained in the NV(*K*_*I*_ + *E*) ensemble. We point out that previous works^17,18^ performing molecular dynamics simulations by using a single Fermi distribution and large electronic temperatures do not fulfill these constraints, as they have a substantially larger violation of the NV(*K*_*I*_ + *E*) ensemble.

For all photoexcitations, the volume *V* is fixed to the ideal volume in the absence of photoexcitation corresponding to the minimum of the equation of state of the GAP potential for Si, i.e., to a relaxed cubic lattice constant of *a* = 5.4716 Å. Volume effects are indeed very small in Si as the minimized volume as a function of the number of photoexcited carriers varies less than 1% with respect to the ground state in both approximations (GAP potentials and full first principles calculation^20^ are perfectly consistent). Incidentally, we recall that it has been shown that the use of a single Fermi distribution with a large electronic temperature (of the order of the laser energy) leads to large errors in the volumes of the photoexcited phases^20^.

We perform MD simulations with a timestep of 0.05 fs for the 7 GAP potentials developed for photoexcited Si at different photocarrier concentrations. The idea is that, even if the electrons are not explicitly included in the calculation, the GAP potential implicitly knows about the forces exerted by the electron-hole plasma on the ions. Consequently, it correctly predicts the ionic dynamics.

**Fig. 5:**
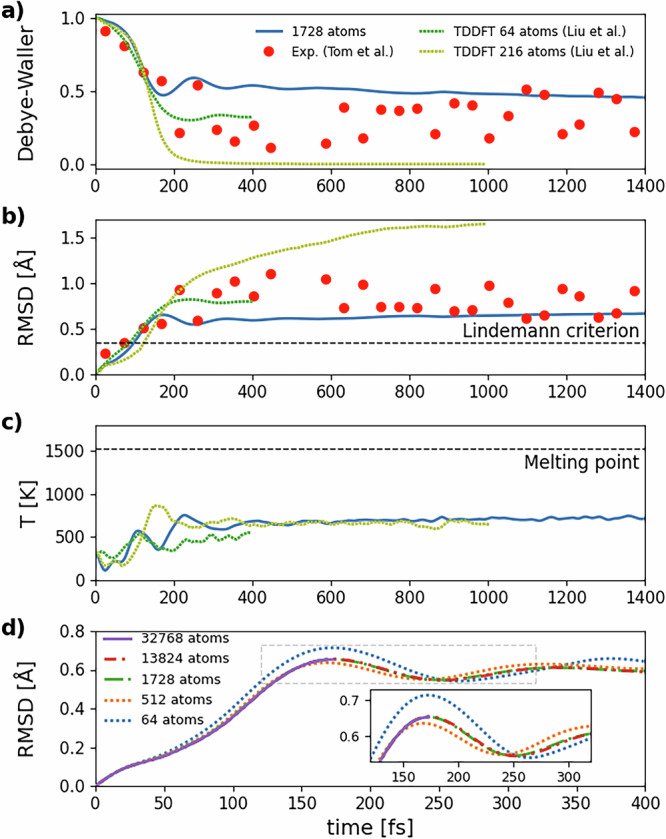
Simulations of non-thermal melting for an initial lattice temperature *T*_*l*_(0) = 300 K with the GAP potential at 0.2 photoinduced electrons/holes per Si atom. **a** Time evolution of the Debye-Waller factor (DW) in the (200) direction, **b** root mean square displacement (RMSD) and (**c**) lattice temperature obtained from a 1728-atom NV(KI + Fel) simulation. Data (blue line) are compared with results from real-time TDDFT by Liu et al.^24^ (green lines) and experiments by Tom et al.^31^ (red points). Experimental RMSD points are obtained from the DW factor applying the inverse of equation (2). Dashed lines show the Lindemann criterion for silicon (0.35 Å) and the theoretical melting temperature of the GAP GS potential (1520 − 1530 K). **d** Dependence of the RMSD shown in (**b**) on system size. Results are reported for simulations with 64, 512, 1728 (same line plotted in (**a**–**c**)), 13824, and 32768 atoms. The inset shows a zoom on the region around the first RMSD peak, highlighted by the dashed gray lines in the main panel. Other data for the larger simulation boxes (up to 32768 atoms) are shown in Supplementary Fig. S5.

In order to validate this educated guess, we set the initial temperature at *T*_*l*_(0) = 300 K and let the ions evolve. Besides the root square mean displacement (RMSD), we also calculate the Debye-Waller (DW) factor

2 $$I(t)={I}_{0}\exp \left[-\frac{{q}^{2}\,\text{RMSD}\,{(t)}^{2}}{3}\right]$$

as already done by Liu et al. In this way we can quickly compare our MD results with both TDDFD and experimental data. In (2), *I*_0_ is an arbitrary intensity normalization and *q* = ∣**q**∣ is the exchanged wavevector between the incoming and outgoing beams in a scattering experiment. We evaluate the DW factor in the direction of reciprocal lattice vector **q** = (200), i.e. **q** = 2**b**_1_, where **b**_1_ is a reciprocal cell vector of the conventional cubic unit cell. Finally, from the ionic kinetic energy, we obtain the lattice temperature as a function of time, i.e. *T*_*l*_(*t*). Results for the GAP potential corresponding to 0.2 photoexcited electrons/holes per Si atom are shown in Fig. 5a–c, where they are compared with experimental data from Ref. ^31^ and with rtTDDFT data with Ehrenfrest dynamics on a 64 and 216 atom simulation box. rtTDDFT data were obtained by considering a 100 − fs pulse of a 610 − nm laser that promotes 11% of valence electrons to the conduction band.

Experimental data were shifted by 100 fs as done by Liu et al. since zero time in experiments was set at the end of a 100 fs-long laser pulse, while *t* = 0 in our idealized MD framework coincides with the arrival of the laser pulse. Finally, experimental data for the DW factor have been normalized by dividing them by the average of data acquired before the laser pulse, i.e. for *t* ≤ −100 fs in Ref. ^31^.

From the behavior of the DW factor and of the RMSD, we see clearly that within the first 100–150 fs after photoexcitation the results of MD with the GAP potential are practically indistinguishable from the rtTDDFT dynamics^24^ and from experimental data. This completely supports our idea that the explicit treatment of the electrons is not necessary if force matching has been performed on the photoexcited electron-hole plasma.

The Lindemann melting criterion states that a material melts when the amplitude of ionic vibrations (represented by the RMSD) reaches a threshold fraction *η* of the bond length. Silicon has a bond length of approximately 2.35 Å and *η* is usually set to 15%, so the Lindemann threshold is 0.35 Å. It is notable that ≈ 100 fs after excitation the Lindemann criterion is already violated and the RMSD is approaching typical values for disordered Si. The lattice temperature also quickly increases. However, it always remains below the melting temperature of solid Si (≈1520–1530 K in our simulations), demonstrating the non-thermal nature of ultrafast melting.

At larger times, from 200 to 400 fs after photoexcitation, we find some discrepancy between rtTDDFT^24^ and our results. Experimental data in the 200 to 400 fs region have a substantial spread, larger than the difference between our simulations and rtTDDFT ones, and are unable to resolve the discrepancy. At times longer than 400 fs, the experimental data for the DW factor have a positive trend that seems to reach our theoretical estimate, although the spread is still large.

We note, however, that rtTDDFT simulations on the largest available simulation box (the cost of rtTDDFT equations limits this technique to a 216-atom simulation box and times smaller than 1 ps) display zero intensity for the DW factor, in stark disagreement with experiments. On the contrary, rtTDDFT simulations on the 64-atom cell, despite the shorter length of the simulation, seem to show a somewhat better agreement with experiment. However, as we believe that the larger simulation box is the one that is more representative of the thermodynamic limit, we believe that this agreement is fortuitous.

Figure 5d shows the dependence of the RMSD on system size for 64, 512, 1728, 13,824, and 32,768 atom cells. Results for 1728 atoms are the same, plotted in panels a–c. The figure shows that simulations for 512 atoms and bigger cells are statistically comparable, since discrepancies between the various results are negligible, so in our approach, 512 atom-systems are already close enough to the thermodynamic limit to provide reliable results. On the contrary, results for 64 atoms deviate from results for bigger cells in a non negligible way. Moreover, for times longer than 200 fs, we find that 64-atom boxes also undergo spurious oscillations in RMSD, DW factor, and *T*_*l*_(*t*) (see Supplementary Fig. S5a–c). These oscillations are quickly suppressed for larger cell simulations.

**Fig. 6:**
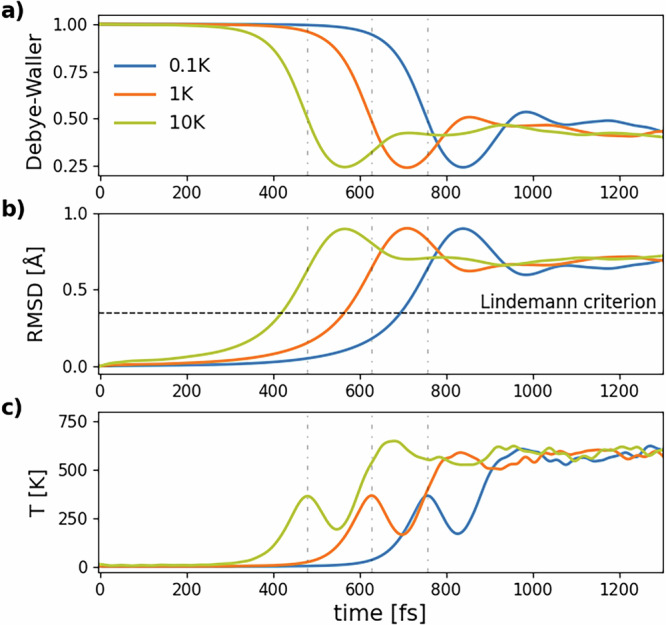
Simulations of non-thermal melting for low initial lattice temperatures with the GAP potential at 0.2 photoinduced electrons/holes per Si atom. Time evolution of (**a**) the DW factor in the (200) direction, **b** the RMSD and (**c**) the lattice temperature for *T*_*l*_(0) = 0.1 K (blue line), *T*_*l*_(0) = 1 K (orange line) and *T*_*l*_(0) = 10 K (green line). Vertical dashed-dotted lines are a guide to the eye to identify the time of the first temperature maxima for each simulation.

**Fig. 7:**
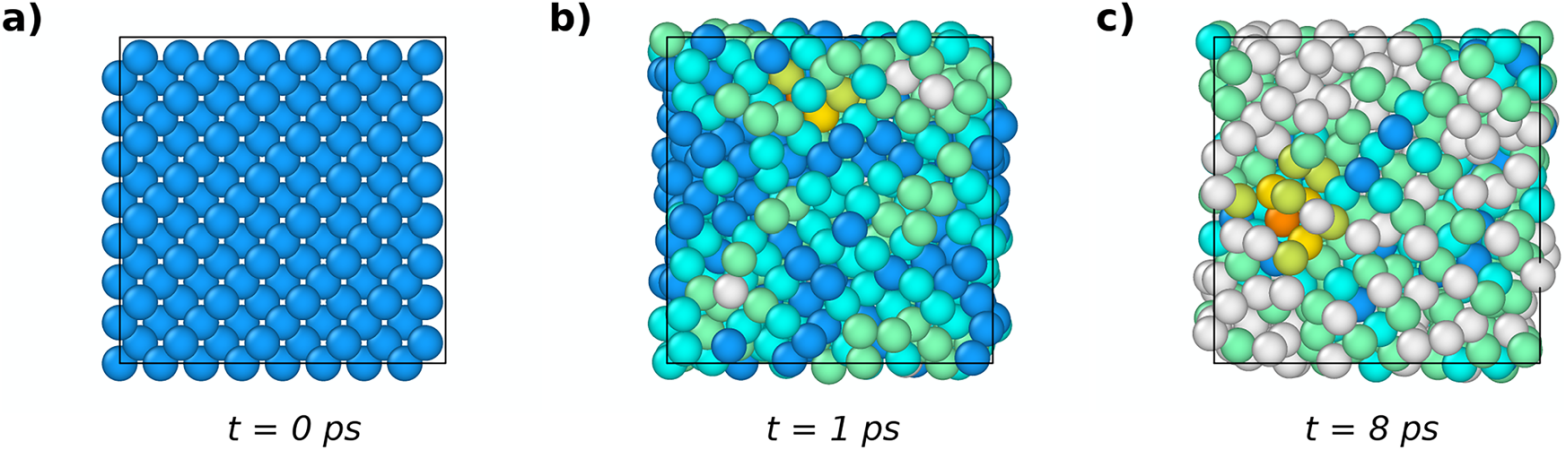
Three snapshots of the 512-atom MD simulation at 0.2 photoexcited electrons/holes per Si atom and *T*_*l*_(0) = 0.1 K, whose DW factor, RMSD, and *T*_*l*_(*t*) are reported in Fig. 6 (blue line). The snapshots are taken at time (**a**) *t* = 0, **b** *t* = 1 ps, i.e., immediately after the first peak in RMSD, and (**c**) *t* = 8 ps, when the system is thermalizing at a temperature *T*_*l*_ ~ 700 K. Images are obtained with the software Ovito^49^. Atoms are colored as follows: blue, light blue, and aqua green refer to atoms with a cubic diamond environment, cubic diamond up to 1st and 2nd neighbors respectively; orange, yellow, and green yellow refers to atoms with a hexagonal diamond environment, hexagonal diamond up to 1st and 2nd neighbors respectively. White means that the atomic environment is none of the above.

Figure 6 shows the results of MD simulations at 0.2 photoexcited electrons/holes per Si atom carried out with different initial lattice temperatures of 0.1 K, 1 K, and 10 K. The time evolution of the RMSD and lattice temperature in all three simulations is very similar except for a time shift. We will show in subsection “Transition modeling and mechanism of non-thermal melting” of section “Results” that this is related to the displacive character of non-thermal melting in the limit of very low lattice temperatures. On the contrary, for higher *T*_*l*_(0), the thermal contribution from ionic velocities becomes relevant. In fact, the timescale for non-thermal melting tends to become smaller for higher temperatures and the first peak in RMSD tends to smear out, as one can see comparing Figs.5 and 6. This behavior is expected as a larger initial temperature means an increased thermal component in the melting process. However, what is unexpected is that such a small temperature increase (from 1 K to 10 K) leads already to a substantial difference in the position of the first peak of the RMSD versus time. Figure 7 shows snapshots of the simulation at *T*_*l*_(0) = 0.1 K in Fig. 6 with identification of the local environment of each atom.

**Fig. 8:**
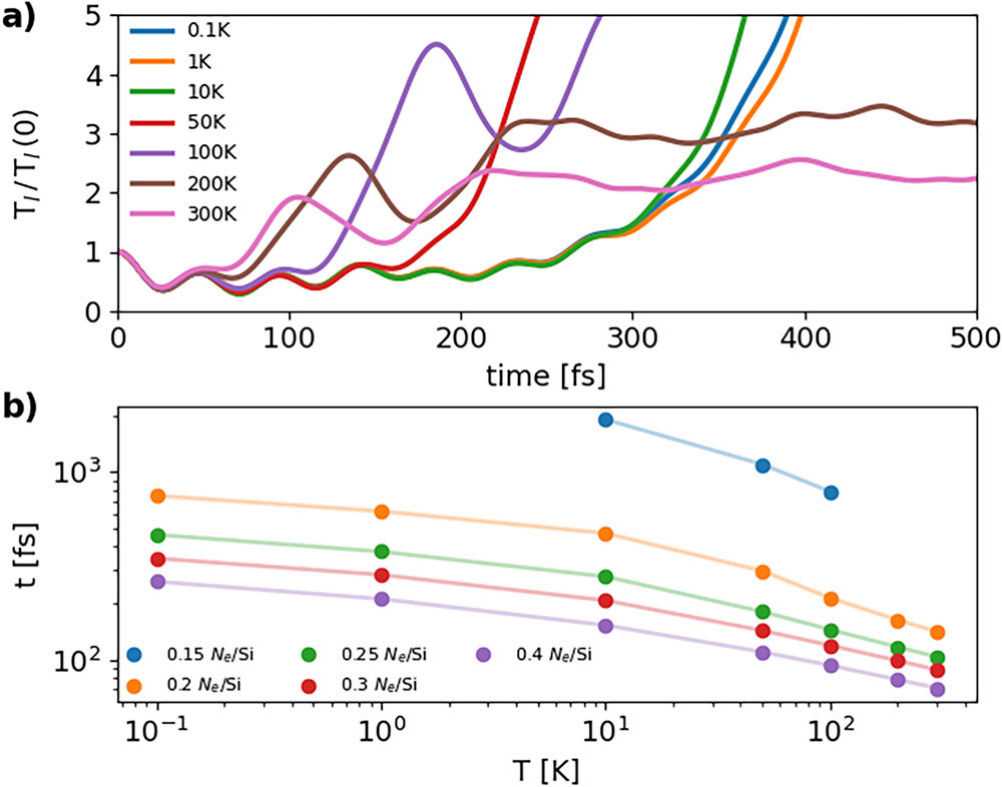
Dependence of non-thermal melting on fluence and *T*_*l*_(0). **a** Normalized temperature *T*_*l*_(*t*)/*T*_*l*_(0) for MD simulations with the potential for 0.2 photoexcited electrons/holes per Si atom. The legend reports the values of *T*_*l*_(0); **b** log-log plot of the time of onset of non-thermal melting as a function of fluence and *T*_*l*_(0) as determined from the Lindemann criterion. Points refer to each MD simulation, lines are a guide to the eye.

Figure 8a shows the normalized lattice temperature *T*_*l*_(*t*) during simulations with different *T*_*l*_(0) and the same potential with 0.2 photoexcited electrons/holes per Si atom. We notice that temperature oscillations occur prior to non-thermal melting (identified by the leading peak in *T*_*l*_(*t*)/*T*_*l*_(0)) and are identical for all temperatures in the first femtoseconds of the simulation. A fit on oscillation maxima and minima gives a frequency *ω* ~ 720 cm^−1^. Since *T*_*l*_(*t*) is proportional to the square of ionic velocities, the frequency of ionic oscillations is half the frequency of temperature oscillations, that is then *ω*/2~360 cm^−1^. The value of *ω*/2 is the energy of the highest energy peak in the phonon DOS in silicon due to optical phonons in Fig. 3. These values for the frequency of temperature oscillations are very similar to those reported by Zijlstra et al.^14^ in ab initio MD of photoexcited silicon at lower fluences below the threshold for non-thermal melting. These oscillations were previously interpreted as squeezed phonons oscillations.

Figure 8a also confirms that the effect of temperature is to activate non-thermal melting earlier. Indeed, for *T*_*l*_(0) → 0 K non-thermal melting occurs after approximately 330 fs, while for increasing temperature it occurs at lower times, down to 50 fs at *T* = 300 K.

Finally, Fig. 8b shows a plot of the time of onset of non-thermal melting as a function of *T*_*l*_(0) and fluence (number of photoexcited electrons/holes per Si atom). The onset of non-thermal melting is measured as the time at which the RMSD reaches the Lindemann criterion (0.35 Å). The figure confirms the observation in Fig. 6 that non-thermal melting becomes macroscopically evident at different timescales. The timescale of non-thermal melting decreases very fast with increasing lattice temperature. The case with 0.15 photoexcited electrons/holes per Si atom has a particular behavior: for very low temperatures (*T* ≤ 1 K), no non-thermal melting is observed at all and the crystal phase is stable. For higher temperatures lattice disordering occurs, but for *T* ≥ 200 K the RMSD never reaches the Lindemann criterion. This anomalous behavior suggests that the threshold photoexcitation for non-thermal melting might be around this value, corresponding to a fluence of 21.2 mJ/cm^2^, as reported in Table 1, and very close to the fluence at which the Kohn anomaly appears, as reported in Fig. 4.

We also notice that non-thermal melting at fixed laser frequency strongly depends on the fluence, i.e., on the percentage of promoted electrons. Non-thermal melting happens on a faster timescale as the fluence increases and generates larger lattice instabilities. As a result of these larger instabilities, the kinetic energy of the atoms after disordering increases. In fact, the final temperature of the system in the NV(*K*_*I*_ + *F*_el_) ensemble reaches up to 2500 K for 0.4 photoinduced electrons/holes per Si atom.

**Fig. 9:**
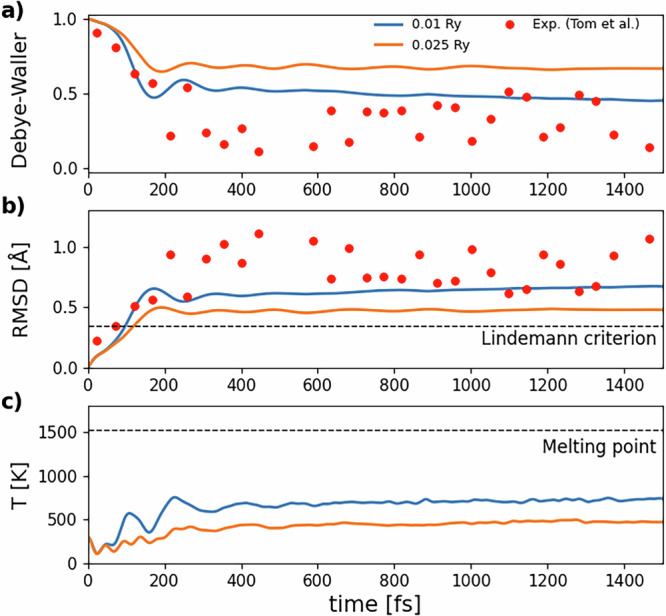
Simulations of non-thermal melting for two electronic temperatures at fixed fluence. Time evolution of (**a**) the DW factor in the (200) direction, **b** the RMSD and (**c**) the lattice temperature obtained with two GAP potentials at 0.2 photoinduced electrons/holes per Si atom with electronic temperatures of 0.01 Ry (blue line) and 0.025 Ry (orange line). MD results refer to NV(*K*_*I*_ + *F*_el_) simulations with 1728 atoms and *T*_*l*_(0) = 300 K. Experimental results by Tom et al.^31^ are also reported (red dots) for a 610 nm laser. Experimental RMSD points are obtained from the DW factor by applying the inverse of equation (2). Dashed lines show the Lindemann criterion for silicon (0.35 Å) and the theoretical melting temperature of the GAP GS potential (1520–1530 K).

Finally, we conclude this section by addressing the role of the electronic temperature *T*_*e*_ used in the fitting of the GAP potential for the solid state of silicon (the dependence on the electronic temperature of the structural properties in the liquid and amorphous phases is very weak). We show in Fig. 9 the comparison between simulations with two different electronic temperatures and experimental data from Ref. ^31^. As it can be seen, a larger electronic temperature generates smaller temperatures in the disordered phases and smaller RMSD, i.e., higher DW factors. In general, as seen in Fig. 9c, the effect of a larger electronic temperature is to reduce the tendency towards melting by smoothing the potential energy surface, and vice versa.

### Transition modeling and mechanism of non-thermal melting

**Fig. 10:**
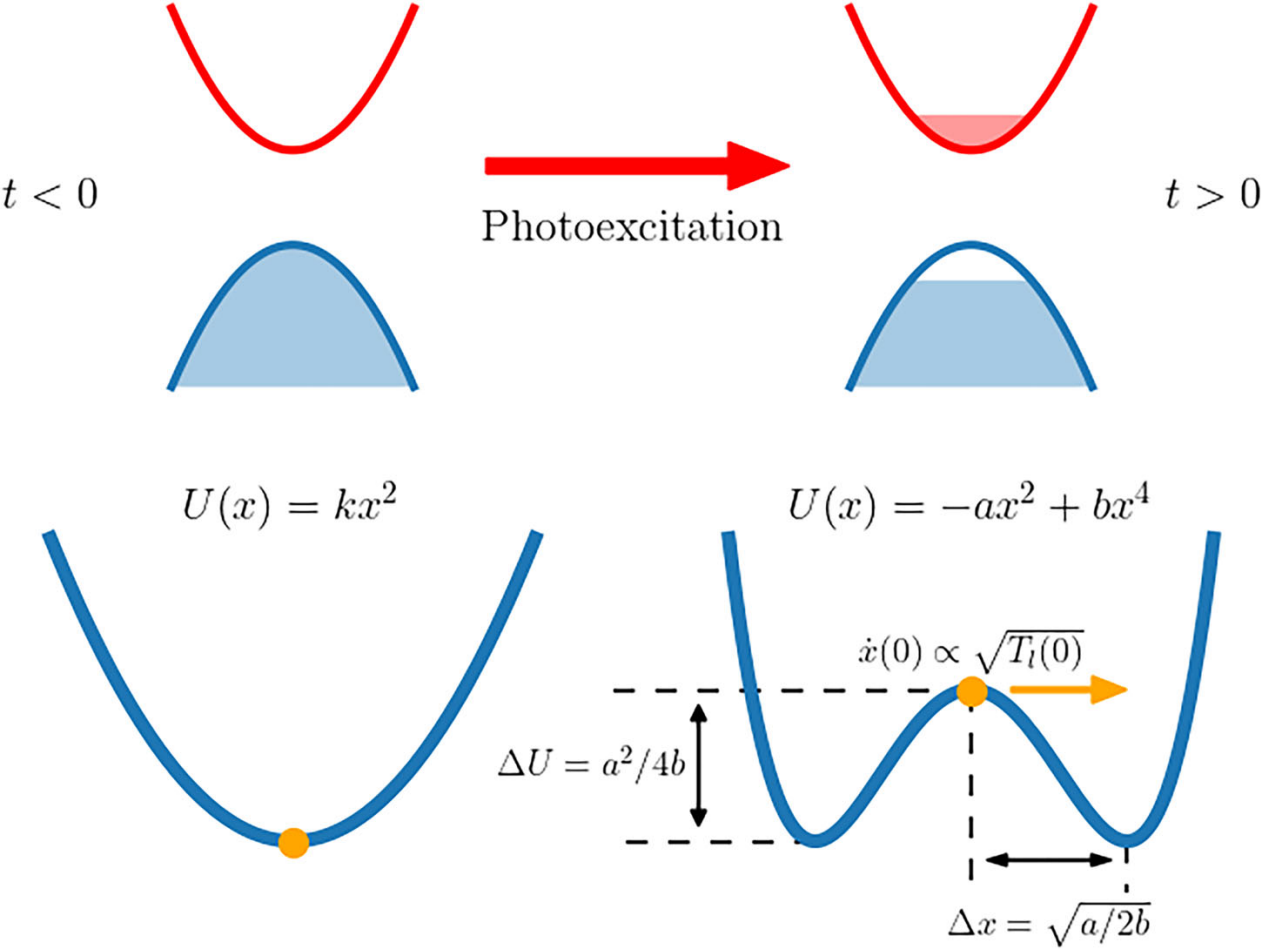
Cartoon of the model employed to describe non-thermal melting, as discussed in the main text. Photoexcitation suddenly changes the potential energy profile in the direction of a phonon mode and introduces an instability, along which the coordinate *x* of the mode will move, starting with an initial velocity proportional to $\sqrt{{T}_{l}(0)}$ by the equipartition theorem. The point of minimum potential energy corresponds with the point of maximum kinetic energy, i.e., with the first peak in lattice temperature in Fig. 6 (highlighted by the vertical dashed-dotted lines). The useful parameters of the quartic potential *Δx* and *ΔU*, used in the main text, are also marked in the figure.

From Fig. 6 we note that the ascending fronts of RMSD(*t*) and *T*_*l*_(*t*) are almost identical for all three simulations, module a shift in time. We guess that this corresponds with the occurrence of a displacive atomic motion along the direction of one (or few) phonon modes. In order to substantiate this statement, we model the results of MD simulations in the following way (see Fig. 10). Before photoexcitation, the system is in its crystalline ground state and all phonon modes are thermalized at the initial lattice temperature *T*_*l*_(0). We can assume that the nuclear system is in the global minimum of its potential energy surface (PES), which can be approximated at first order as a harmonic function (left part of Fig. 10). Then, photoexcitation and electron intraband thermalization occur. We can assume both processes as instantaneous with respect to the ionic motion and, thus, occurring at time *t* = 0 of the MD simulation. However, they abruptly change the PES of the system, that suddenly finds itself in a local maximum in phase space (right part of Fig. 10). In other words, photoexcitation has created an instability via a soft phonon (displacive phase transition).

As a consequence, the system moves towards the new potential energy minimum. The initial lattice temperature *T*_*l*_(0) determines the initial velocity of this motion, which is why the timescale for non-thermal melting tends to increase as *T*_*l*_(0) decreases. For simplicity, we can assume that the new minimum in the PES is created along a direction in phase space specified by a single phonon eigenvector, as schematized in Fig. 10. However, even if the direction were specified by a linear combination of phonon eigenvectors, the same treatment would still hold.

The motion of the system along the chosen direction in phase space can be modeled as a one-dimensional problem, where an effective particle of mass *m* moves in a potential energy *U*(*x*), being *x* the coordinate that measures displacement with respect to the non-photoexcited equilibrated system along the chosen direction in phase space. Before photoexcitation, i.e., for *t* < 0, the potential energy can be assumed to be a harmonic function *U*(*x*) = *kx*^2^/2, and the particle is located in its minimum, that is *x* = 0. Immediately after photoexcitation, for *t* ≥ 0, due to laser-induced phonon instabilities, the potential can be described with a quartic potential of the type

3 $$U(x)=-a{x}^{2}+b{x}^{4},$$

with *a*, *b* > 0. The initial position of the particle is still *x* = 0, since photoexcitation has changed the PES in an extremely short time with respect to the ionic motion. Now, *x* = 0 is the local maximum of the potential, since − *a* < 0. The initial velocity $\dot{x}(0)$ of the phonon mode at *x* = 0 is also identical to the one before photoexcitation and is obtained from the equipartition theorem, assuming that the phonon population is thermalized at the same lattice temperature *T*_*l*_(0) imposed at the start of the MD simulation:

4 $$\frac{1}{2}m{[\dot{x}(0)]}^{2}=\frac{1}{2}{k}_{B}{T}_{l}(0),\,\,{\text{hence}}\,\,{\dot{x}}(0)=\sqrt{\frac{{k}_{B}}{m}{T}_{l}(0)},$$

where *k*_*B*_ is the Boltzmann constant.

Due to the initial velocity $\dot{x}(0)\ne 0$, the particle will start to move in the potential, reaching one of the two (symmetric) minima located at $| {x}_{m}| =\sqrt{a/2b}$ and then undergo a periodic motion. We are interested in estimating the total time *Δt* taken by the particle to reach the minimum of the potential energy *U*(*x*), which corresponds to the condition of maximum kinetic energy and minimum potential energy in the MD simulation. This time corresponds with the time *Δt* taken by the MD system to reach the first peak in *T*_*l*_(*t*) highlighted by the dashed-dotted lines in Fig. 6. A rough estimate allows to conclude that, although the value of *Δt* depends on *T*_*l*_(0), the temperature at the first peak has approximately the same value *T*_*l*_(*Δt*) ~ 365 K for all three MD simulations. If at small times the motion of the MD system occurs only in one single effective direction, the RMSD given by MD should correspond with the displacement *Δx* of the effective particle in the one-dimensional model. For this reason, the distance between the initial position *x* = 0 and the potential minimum, defined as $\Delta x=| {x}_{m}| =\sqrt{a/2b}$, should correspond with RMSD(*Δt*) from the MD trajectory. Figure 6 confirms this idea, since the RMSD has approximately the same value RMSD(*Δt*) ~ 0.636 Å, for all three simulations.

The equation of motion for the model phonon mode is

5 $$\frac{{d}^{2}x}{d{t}^{2}}=-\frac{1}{m}\frac{dU}{dx}=\frac{2a}{m}{x}^{2}-\frac{4b}{m}{x}^{3},$$

The total displacement can then be written as

6 $$\Delta x=\sqrt{a/2b} \sim 0.636\,{\mathring{\rm{A}}} ,$$

that provides a condition on the values of *a* and *b*. A second condition on *a* and *b* comes from the fact that the total variation of kinetic energy of the particle in our model is equivalent to the increase in kinetic energy of the MD system, that can be related to its lattice temperature *T*_*l*_(*t*). Assuming *T*_*l*_(0) ≪ *T*_*l*_(*Δt*), that is true by almost two orders of magnitude, the total variation of kinetic energy of the particle in our model corresponds with the kinetic energy at time *t* = *Δt* in MD trajectories. This variation in kinetic energy is equal to minus the total variation of potential energy of the effective particle in the potential *U*(*x*) between time *t* = 0 and *t* = *Δt*. This last quantity can be found as the difference between the value of *U*(*x*) at the local maximum and at the minimum, i.e. $\Delta U=U(0)-U(\pm \sqrt{a/2b})$, and is given by

7 $$\Delta U=\frac{{a}^{2}}{4b} \sim \frac{1}{2}m{\left[\dot{x}(\Delta t)\right]}^{2}=\frac{1}{2}{k}_{B}{T}_{l}(\Delta t).$$

Inverting the relation and using *T*_*l*_(*Δt*) ~ 365 K one obtains

8 $${a}^{2} \sim 4\Delta Ub \sim \left(6.069\times 1{0}^{-4}\,\,\text{amu}\,\,{{\mathring{\rm{A}}} }^{2}/f{s}^{2}\right)b,$$

where we have chosen to express masses in amu, lengths in units of Å and times in fs. Considering equations (6) and (8), we obtain a system of two equations in two variables for *a* and *b*, which can be solved and give us some estimates for the parameters that enter differential equation (5). The resulting numerical values are *a* ~ 7.503 × 10^−4^ amu/fs^2^ and *b* ~ 9.274 × 10^−4^ amu/(Å^2^f*s*^2^). These values are independent of the effective mass *m* that is chosen, as expected for frictionless motion.

**Fig. 11:**
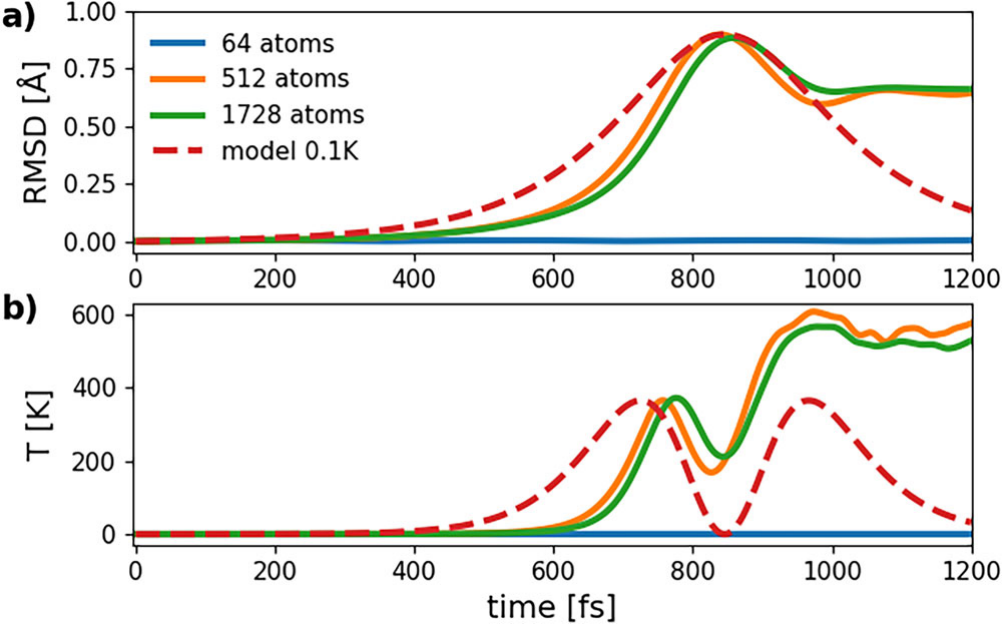
Comparison between MD simulations and the one dimensional model introduced in Fig. 10. **a** RMSD as obtained from the one dimensional model and from MD simulations at 0.2 photoexcited electrons/holes per Si atom with an initial lattice temperature *T*_*l*_(0) = 0.1 K; **b** lattice temperature as obtained from the one dimensional model and from the same MD simulations.

We can now solve the differential equation (5) with the estimated values of *a* and *b* and with initial conditions *x*(0) = 0 and $\dot{x}(0)=\sqrt{{k}_{B}{T}_{l}(0)/m}$, respectively. The result is shown in Fig. 11. The dashed red line shows the effective displacement *x*(*t*) and effective lattice temperature ${T}_{m}(t)=m{[\dot{x}(t)]}^{2}/{k}_{B}$ obtained from the one-dimensional model with an initial temperature *T*_*l*_(0) = 0.1 K. The figure also reports the RMSD and lattice temperature from three MD simulations at *T*_*l*_(0) = 0.1 K with identical parameters but different system sizes (64, 512, and 1728 atoms). The plots for 512 and 1728-atom systems are very similar and confirm that a system size of 512 atoms is already very close to the thermodynamic limit. On the contrary, results for the 64-atom system are totally different. In fact, both RMSD(*t*) and *T*_*l*_(*t*) remain very low for the whole simulation time that is considered. This means that with a 64-atom system non-thermal melting is not observed at *T* = 0.1K, suggesting that phonon modes involved in the disordering of the lattice are not commensurate with a 2 × 2 × 2 supercell.

The agreement between MD results and the one-dimensional soft phonon model up to the first peak in RMSD is impressive and confirms that non-thermal melting, at least for low lattice temperatures, is well described by a displacive phase transition due to a soft phonon instability that arises in the PES. This is at odds with the thermal melting process, which is a first-order phase transition.

For *t* > *Δt*, agreement with the model is lost as the MD trajectory becomes more complex and significant contributions from all phonon modes are present.

This analysis can be brought further by identifying the phonon mode that is responsible for the insurgence of non-thermal melting. Previous considerations on system size suggest that the phonon mode driving the transition is imaginary only for supercells larger or equal than a 4 × 4 × 4 supercell of the cubic conventional unit cell (512 atoms) while its frequency is positive and real throughout the full zone in a 2 × 2 × 2 supercell (64 atoms). To better understand this behavior, we compute the harmonic phonons of silicon with QE on a *N* × *N* × *N* q-point grid, with *N* = 2, 4, 6, using the 8-atom cubic unit cell, the same pseudopotential, and the same values for all other parameters as used in previous calculations. In this way, we obtain the Cartesian components of the phonon eigenmodes ${e}_{{\bf{q}}\nu }^{c\gamma }$ from first principles. We label atoms in the unit cell with *c* = 1, . . . , *N*_*u*_, the Cartesian components of the displacement of each atom with *γ* = *x*, *y*, *z*, the phonon wavevector with **q**, and its band index with *ν* = 1, . . ., 3*N*_*u*_. The number of independent **q** wavevectors is *N*^3^. The total number of phonon modes is then 3*N*_*u*_*N*^3^, which is also equal to the dimensionality of the configuration space of the MD system built as a *N* × *N* × *N* supercell of the 8-atom conventional unit cell, that contains *N*_*u*_*N*^3^ atoms.

Then, we consider the MD trajectory on the corresponding *N* × *N* × *N* supercell. We call *r*_*aα*_(*t*) the Cartesian component *α* of the position of atom *a* in the MD cell at time *t*. We define

9 $$\Delta {r}_{a\alpha }(t)=\frac{{r}_{a\alpha }(t)-{r}_{a\alpha }(0)}{{\left[\mathop{\sum }\nolimits_{b = 1}^{{N}_{u}{N}^{3}}{\sum }_{\beta }{\left({r}_{b\beta }(t)-{r}_{b\beta }(0)\right)}^{2}\right]}^{1/2}},$$

that is the MD displacement of Cartesian component *α* of atom *a* between time *t* and time 0. Here, *α*, *β* = *x*, *y*, *z* and *a*, *b* = 1, . . . , *N*_*u*_*N*^3^. The displacement is normalized so that the vector ${\{\Delta {r}_{a\alpha }(t)\}}_{a\alpha }$ has norm one for all times. The contribution of each phonon mode to the MD trajectory *Δr*_*aα*_(*t*) is intuitively given by the scalar product between the phonon eigenmodes ${e}_{{\bf{q}}\nu }^{c\gamma }$ and the normalized MD trajectory *Δr*_*aα*_. However, the eigenvectors ${e}_{{\bf{q}}\nu }^{c\gamma }$ are defined only on the unit cell, while *Δr*_*aα*_(*t*) is defined on the *N* × *N* × *N* supercell. In order to compare the two vectors, we compute the displacement on all atoms in the supercell as induced by the eigenmode ${e}_{{\bf{q}}\nu }^{c\gamma }$. The displacement on corresponding atoms in different copies of the unit cell will be identical module a phase shift given by the **q** vector of the mode:

10 $${f}_{{\bf{q}}\nu }^{a\alpha }={e}_{{\bf{q}}\nu }^{u(a)\alpha }{e}^{i{\bf{q}}\cdot {{\bf{R}}}_{v(a)}},$$

where *u*(*a*) is a mapping function that maps atom *a* = 1, . . . , *N*_*u*_*N*^3^ in a generic copy of the unit cell to its corresponding image *c* = 1, . . . , *N*_*u*_ in the unit cell and *v*(*a*) is a function that maps atom *a* to the index of its unit cell. Thus, **R**_*v*(*a*)_ corresponds to the direct lattice vector that identifies the unit cell in which atom *a* lies. The ordering of atoms in ${f}_{{\bf{q}}\nu }^{a\alpha }$ is the same as in *Δr*_*aα*_(*t*). Last, we renormalize ${f}_{{\bf{q}}\nu }^{a\alpha }$ so that it has norm 1.

The value of the scalar product between the eigenmode and the normalized MD trajectory is then given by

11 $${\eta }_{{\bf{q}}\nu }(t)=\left\langle f| \Delta r(t)\right\rangle =\mathop{\sum }\limits_{a=1}^{{N}_{u}{N}^{3}}\sum _{\alpha }{f}_{{\bf{q}}\nu }^{a\alpha }\Delta {r}_{a\alpha }(t)$$

Intuitively, *η*_**q***ν*_(*t*) gives an idea of how much mode **q***ν* contributes to the total MD displacement. This procedure corresponds to decomposing the normalized MD trajectory in configuration space in the orthonormal complete basis of the phonon eigenmodes. If non-thermal melting is due to instabilities in the potential energy surface induced by photoexcitation, this means that MD displacements should have a very big overlap with the few phonon eigenmodes that are associated to imaginary frequencies *ω*_**q***ν*_ < 0 in the ab initio phonon calculations. Thus, we split the contributions of each phonon mode to MD displacements between those with real and imaginary ab initio frequencies:

12 $${\eta }_{{\rm{real}}}(t)={\left[\sum _{{{\bf{q}}}{\nu }:{\omega }_{{\bf{q}}\nu }\ > \ 0}{\left\vert {\eta }_{{\bf{q}}\nu }(t)\right\vert }^{2}\right]}^{1/2}$$

and

13 $${\eta }_{{\rm{imag}}}(t)={\left[\sum _{{{\bf{q}}}{\nu }:{\omega }_{{\bf{q}}\nu } < 0}{\left\vert {\eta }_{{\bf{q}}\nu }(t)\right\vert }^{2}\right]}^{1/2}.$$

The normalization condition is

14 $${\eta }_{\,\,\text{real}\,}^{2}(t)+{\eta }_{\,\,\text{imag}\,}^{2}(t)=\parallel \Delta {r}_{a\alpha }(t){\parallel }^{2}=1.$$

We notice that *η*_ real_(*t*) and *η*_ imag_(*t*) are invariant by a phase transformation (independent of the atomic position) of the eigenvectors.

**Fig. 12:**
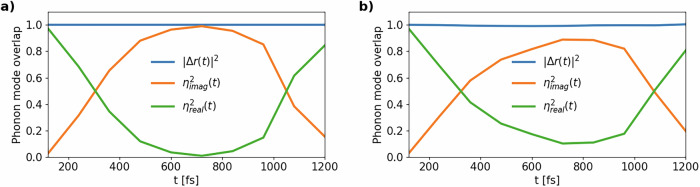
Decomposition of the normalized MD displacements with respect to real and imaginary phonon eigenmodes of the cubic conventional unit cell of silicon. The two contributions *η*_ real_(*t*) and *η*_ imag_(*t*) are defined as in Eqs. (12) and (13), respectively, and represent the modulus of the overlap between the MD normalized trajectory and the phonon eigenvectors of real and imaginary phonon modes, respectively. The MD simulations considered are (**a**) the 512-atom one and (**b**) the 1728-atom one at *T*_*l*_(0) = 0.1 K with the GAP potential for 0.2 photoexcited electrons/holes per Si atom. The overlap is shown for 10 times, ranging between 0 and 1.2 ps, i.e. until both the RMSD and lattice temperature stabilize after non-thermal melting has occurred. Imaginary modes account for 97% and 87% of the MD normalized displacements for *t* ~ 700 fs, respectively.

We compute *η*_ real_(*t*) and *η*_ imag_(*t*) for the MD trajectory at *T*_*l*_(0) = 0.1 K with 512 atoms and for a set of 10 times in the range [0, 2*Δt*]. The result is reported in Fig. 12. For the 512-atom system, there are 36 imaginary and 1500 real modes. Thus, imaginary modes are just 2.3% of the total and are all acoustic modes at intermediate points of the BZ of the cubic cell, that are the same points that have instabilities in the harmonic phonon spectrum of the two-atom primitive unit cell shown in Fig. 3. Noticeably, no q-point commensurate with the 2 × 2 × 2 supercell has instabilities nor a relevant contribution to the MD displacements, confirming our observation that 64-atom systems are too small to show non-thermal melting in the low-temperature regime. This commensurability barrier is probably removed by thermal motion of atoms at room temperature, providing both us and Liu et al. with evidence of non-thermal melting in 64-atom systems with higher *T*_*l*_(0).

At the start of the 512-atom simulation, the contribution to MD displacement is uniformly distributed between hundreds of modes. This is due to the random initialization of velocities. However, as time passes, the contribution to MD displacements by imaginary modes grows more and more, until accounting for up to 97% of MD displacements for *t* ~ *Δt*, i.e. ${\eta }_{\,\,\text{imag}\,}^{2}(t) \sim 0.97$. This means that imaginary modes only are driving the system towards non-thermal melting and they are responsible for RMSD and temperature increase. This confirms the validity of the one-dimensional model developed to describe MD trajectories. After non-thermal melting, the model does not hold anymore; in fact *η*_ imag_(*t*) drops very quickly to small values.

We remind that Fig. 3 shows the harmonic phonons of photoexcited silicon at 0.2 e^−^/atom, while MD displacements effectively include anharmonic corrections up to all orders. The fact that an anharmonic estimate of phonon instabilities in photoexcited silicon from MD trajectories gives similar results to ab initio harmonic results is a hint that anharmonic corrections to the latter are not sufficiently large to cure the phonon instabilities.

To check the impact of the initial velocity distribution, we run another MD simulation with a different random seed for the initialization of atomic velocities at time *t* = 0. The resulting plot does not present significant differences with respect to the one in Fig. 12 and is not reported here.

The analysis of a 1728-atom MD trajectory confirms all the main observations discussed above: non-thermal melting arises again from a limited number of acoustic phonon modes that have imaginary harmonic frequencies. In this case, the total number of modes is 5184, of which 120 are imaginary. The percentage of imaginary modes is notably still 2.3%. At *t* ~*Δt*, the total contribution to non-thermal melting from imaginary modes reaches 87%, i.e. ${\eta }_{\,\,\text{imag}\,}^{2}=0.87$.

We conclude this Section with a couple of remarks. First, the results of this work do not include quantum anharmonicities since MD simulations provide classical results. Second, we are not aware of experimental measurements of non-thermal melting at low temperatures, which could provide very valuable information about its origin. Last, the 1D model is valid in the limit *T*_*l*_(0) → 0 and for low enough fluences. For higher temperatures like *T*_*l*_(0) = 300 K (Fig. 5) the 1D model is not sufficient to describe non-thermal melting since thermal effects due to ionic velocities also enter the game and the interplay between them is difficult to disentangle. If the fluence is increased too much above the critical value for non-thermal melting, the sound velocity at *Γ* becomes negative (Fig. 4), and again the character of the phase transition changes.

Supplementary Section S10 contains a discussion about the applicability of this model to other materials.

## Discussion

In this work we developed a machine learning approach to perform highly efficient simulations without loss of accuracy with respect to ab initio molecular dynamics. The method and its novelty rely on two key ingredients. The first is the constrained density functional perturbation theory method developed in Ref. ^20^ that allows for very accurate modeling of structural and vibrational properties in the presence of a photoinduced electron-hole plasma in the case when electron-hole recombination is slower than the structural transition induced by the laser. The second is a definition of the training set leading to machine learning potentials that are able to accurately describe the crystalline and disordered phases as well as the melting of silicon both in the thermal and non-thermal case. These developments are completely general and do not rely on a specific kind of machine learning approach.

We apply the method to non-thermal melting in silicon and compare the results with recent rtTDDFT simulations. From the comparison, we draw two main conclusions. First, in the initial 150 fs after laser pumping, the two approaches yield very similar results, suggesting that the explicit treatment of electrons is not relevant for the physics of non-thermal melting. The role of the time evolution of the electronic temperature(s) during the simulation is discussed in Supplementary Section S7. Thus, we believe that the approach presented here represents a valuable and considerably cheaper alternative to rtTDDFT simulations. Second, above 150 fs, the rtTDDFT simulations on the largest available cells show zero intensity for the Debye-Waller factor along the (200) direction, in stark disagreement with experiments. Our simulations on the largest simulation box (1728 atoms) and at times as long as 1400 fs are in better agreement with the Debye-Waller factor measured in experimental data, although the latter are fairly scattered.

For the specific case of silicon, we developed a one-dimensional model that closely captures the essence of non-thermal melting. The model, the interpretation of machine learning molecular dynamics simulations and its comparison with cDFPT phonon dispersion demonstrate that at the fluences considered in our work, non-thermal melting occurs via a soft phonon, at odds with thermal melting, that is a strictly first order phase transition with no phonon softening.

Our simulations also account for a certain number of phenomena. For example Fig. 8 shows that the higher the fluence, the faster non-thermal melting is, in agreement with experiments (see Fig. 4 in ref. ^8^). Moreover, the larger the initial temperature before laser pumping, the faster the melting (see Fig. 6).

In conclusion, the generality of the proposed framework makes it easily exportable to other problems, such as the study of photoinduced disordering of vanadium dimers in VO_2_ or non-thermal phase transitions in other semiconductors, phase change materials, and ferroelectrics. The obtained accuracy, comparable with the of other ab initio approaches but considerably faster, demonstrates the possibility to perform large-scale simulations of photoexcited systems for longer times, opening to the systematic investigation of light-driven order-disorder phase transitions.

## Methods

### Ab initio calculations

Energies, forces and virial stresses are computed from first principles using the publicly available code QUANTUM ESPRESSO^36–38^ (QE) version 7.2, that includes the two Fermi level approach developed in ref. ^20^. Calculations are performed using the Pedrew-Burke-Ernzerhof (PBE) approximation^39^ for the exchange-correlation energy within the generalized gradient approximation (GGA) framework. Core electrons are described through the ultrasoft^40^ pseudopotential Si.pbe-van_gipaw.UPF from http://www.quantum-espresso.org with the suggested plane wave cutoffs of 18.372 Ry for the energy and 249.156 Ry for the density. We employ a uniform k-point density of spacing 0.17 Å^−1^ and total energy convergence threshold of 10^−6^ Ry. The specific values of electronic temperature used to generate the various datasets are discussed in the next paragraph. Phonon calculations with the two Fermi level approach are performed with QE version 7.4.

### GAP fitting

A general-purpose GAP potential by Bartók et al. is already publicly available for ground state, i.e., non photoexcited, silicon^29^. The dataset consists of 2475 configurations including bulk crystalline, liquid, and amorphous phases, as well as vacancies, surfaces, interstitial atoms, dislocations and cracks. As we are interested in describing bulk properties of silicon, we only retain configurations from the liquid and amorphous phases, as well as the 4 lowest-energy crystalline structures, namely fcc diamond, hexagonal diamond, bc8, and st12. This leads to the 872-configuration dataset described in the left side of Table 2.

**Table 2:** List of configurations used for training GAP potentials

Structure type	No. atoms	No. structures	*σ*_energy_ [meV/atom]	*σ*_force_ [eV/Å]	*σ*_virial_ [eV/Å^3^]
Isolated atom	1	1	0.001	0.01	0.05
Diamond	2	104	0.001	0.01	0.05
	16	220			
	54	110			
	128	55			
Liquid	64	69	0.003	0.15	0.2
	128	7			
Amorphous	64	31	0.01	0.2	0.4
	216	128			
Hex. diamond	4	49	0.001	0.01	0.05
Bc8	8	49	0.001	0.01	0.05
St12	12	49	0.001	0.01	0.05

All configurations come from the database by Bartók et al.^29^ and have been recomputed with the pseudopotential of interest. The first column shows the various phases included in the dataset, the second column shows the number of atoms in each structure, and the third column shows the number of structures of that type in the database. The total number of structures in the database is 872. For photoexcitations above 0.1 *N*_*e*_/Si, the crystal structures in the last three lines are removed from the database, yielding a dataset of 725 structures. The last three columns report the regularization parameters for each structure type used in GAP fitting for the energy, the forces, and the virial, respectively. We used the same regularization values as Bartók et al., apart from *σ*_force_ for crystalline phases, which was reduced by a factor of 10 to improve accuracy in the phonon dispersion of the bulk diamond phase.

As the potential energy surface is substantially dependent on the number of photoexcited electrons/holes and on the electronic temperature, we verify that substantially different potentials are needed for different fluences. Thus, we generate 9 different datasets and fit 9 different GAP potentials for 8 different fluences (including one potential in the absence of photoexcitation and two potentials for the same fluence, but for different electronic temperatures). We use the QUIPPY package^41^ version 0.9.9. We use a smooth overlap of atomic position (SOAP) kernel^42^, and we retain the same two-body repulsive baseline used by Bartók et al. to improve fit stability.

For each dataset, we perform a search in hyperparameter space looking for values of the hyperparameters that give a good tradeoff between accuracy and computational cost. We evaluate the quality of fits by looking at root mean square errors (RMSEs) on energy, forces, and virial stress in the training set. Results for potentials in the presence of photoexcitation are comparable to those obtained in the absence of photoexcitation. This shows that our potentials manage to learn the relevant features of the potential energy surface in the presence of photoexcitation.

The optimal values of the hyperparameters are given in Table 3 for (i) the ground state (no photoexcited electrons), (ii) the 7 photoexcited potentials as a function of the amount of photoexcited electrons/holes with an electronic temperature of of *T*_*e*_ = 0.01 Ry = 1579 K and (iii) an auxiliary potential corresponding to 0.2 photoexcited electrons/holes per Si atom and a larger electronic temperature of *T*_*e*_ = 0.025 Ry = 3947 K. The structure-specific regularization parameters are the same for all GAP fits and are reported on the right side of Table 2.

**Table 3:** Electronic temperatures used in QE calculations and GAP hyperparameters

*N*_*e*_/Si	*T*_*e*_ (K) [gapped]	*T*_*e*_ (K) [non-gapped]	Cutoff [Å]	*n*_sparse_	${n}_{\max }$	${l}_{\max }$
0	1579	1579	5.0	12,000	12	10
0.05	1579	395	4.0	6000	10	6
0.1	1579	790	5.0	9000	12	10
0.15	1579	1184	5.5	12,000	14	12
0.2	1579	1579	5.5	12,000	12	12
0.25	1579	1974	5.5	12,000	12	12
0.3	1579	2369	5.5	12,000	12	12
0.4	1579	3158	6.0	12,000	14	12
0.2	3947	1579	5.5	12,000	12	12

List of electronic temperatures used in QE calculations for photoexcited gapped and non-gapped configurations (second and third column, respectively) as a function of fluence (first column). Columns from the fourth on show the optimal hyperparameters used to fit GAP potentials for each database. Optimality is judged from energy, force, and virial RMSEs, and from the accuracy of the phonon dispersion. Starting from 0.1 *N*_*e*_/Si, higher-energy crystalline phases (hexagonal diamond, bc8, and st12) have been excluded from the database.

We used Fermi-Dirac type smearing for all QE calculations and divided the original database by Bartók et al.^29^ between gapped and non-gapped configurations using a threshold of 0.2 eV in the electronic density of states. This classifies all crystalline configurations in our dataset as gapped and all disordered configurations as non-gapped. Photoexcited gapped configurations treated with the two Fermi level approach were computed with the values of *T*_*e*_ discussed above and reported in the second column of Table 3. For non-gapped configurations treated with the single Fermi level approach, the electronic temperature was increased linearly with the number of excited carriers in the corresponding gapped phases and is reported in the third column of Table 3. Intuitively, increasing the amount of excited carriers increases the excess energy that ultimately relaxes to the lattice after electron-ion recombination. However, different electronic temperatures in non-gapped disordered phases have a negligible impact on the insurgence of non-thermal melting in MD simulations, as well as on structural properties of disordered phases like radial distribution functions.

### Molecular dynamics simulations

We perform all MD simulations in this work with the LAMMPS software version 28 March 2023^43^ patched with the ML-QUIP package. Interface simulations to estimate the melting temperature of the GAP GS potential are performed by adapting the method described by Morris and Song^44^. More details about MD simulations, the role of the electronic temperature^45^ and a discussion about the NV(*K*_*I*_ + *F*_el_) and NV(*K*_*I*_ + *E*) ensembles are contained in Supplementary Section S2. Additional considerations about the electronic temperature are reported in Supplementary Section S7^46^.

## Supplementary information

Supplementary information

## Data Availability

All data relevant to the reproducibility of this work (input and output files, software versions, data analysis scripts and data for all the Figures) will be made publicly available on Zenodo (10.5281/zenodo.14916551) upon article publication^47^.
