# Supplementary material for: Scalable machine learning approach to light induced order disorder phase transitions with ab initio accuracy
Source: NPJ Comput Mater. 2025 May 26;11(1):151. doi: 10.1038/s41524-025-01614-5 (PMC12106065; doi:10.1038/s41524-025-01614-5)
Supplement: Supplementary file 1 — Supplementary information [file 41524_2025_1614_MOESM1_ESM.pdf]

Supplemental Material for "Scalable machine learning approach to light induced order disorder phase transitions with *ab initio* accuracy".

Andrea Corradini,[1] Giovanni Marini,[1] and Matteo Calandra[1]

[1] *Department of Physics, University of Trento, Via Sommarive 14, 38123 Povo, Italy*

S1. FORMULA FOR THE RELATIVE ERROR

The relative error on forces has been computed as

$$\eta = \sqrt{\frac{\sum_{i=1}^{N_c} \sum_{j=1}^{a(i)} \left| \mathbf{f}_{i;j}^{\text{GAP}} - \mathbf{f}_{i;j}^{\text{DFT}} \right|^2}{\sum_{i=1}^{N_c} \sum_{j=1}^{a(i)} \left| \mathbf{f}_{i;j}^{\text{DFT}} \right|^2}}, \tag{1}$$

as done by Bauerhenne *et al.*[1] and Plettenberg *et al.*[2]. Here the summation index $i = 1, ..., N_c$ runs over configurations in the training set and $j = 1, ..., a(i)$ over the atoms in a given configuration. $\mathbf{f}_{i;j}$ is then the total force on atom j in configuration i. This formula can be easily adapted to the energy or virial by removing the summation over atoms and replacing $\mathbf{f}_{i;j}$ with the scalar cohesive energy or virial tensor for the given configuration.

Overall, our GAP potentials have an electronic temperature and fluence independent relative error of $0.02 - 0.06\%$ on the average energy, $13.1 - 16.6\%$ on the average force and $3.0 - 4.4\%$ on the average virial. We remind that the analytical potential in Ref. [1] claims an accuracy of 26% on the average force at small electronic temperatures and an accuracy of 6% at very high electronic temperatures, where the potential energy surface is far less complex; the Behler-Parrinello neural network potential by Plettenberg *et al.*[2] has relative error between 8% and 16% on forces and between 0.1% and 15% for energies in the whole range of electronic temperatures considered, as shown in Fig. 2 in their work. Thus, our GAP potentials have approximately the same accuracy of previous state-of-the-art works[1, 2] in force estimation. In energy estimation, our potentials perform much better than previous potentials available in literature.

S2. LAMMPS SIMULATIONS

A. Mermin free energy and NV$(K_I + F_{\text{el}})$ ensemble

As already stated in the main text, we run MD in the microcanonical ensemble and the conserved energy is the sum of the nuclear kinetic energy K_I and the electronic Mermin free energy $F_{\text{el}} = E - TS$, where E is the internal energy and T and S are respectively the temperature and entropy of the electronic Fermi gas. The entropy of a Fermi gas is given by

$$S = -k_B \sum_i \left[f_i \ln f_i + (1 - f_i) \ln(1 - f_i) \right], \tag{2}$$

where f_i is the occupancy of state i and the summation runs over all available electronic states. In the case of the two Fermi level treatment, the Mermin free energy correspondingly reads $F_{\text{el}} = E - T_{\text{val}} S_{\text{val}} - T_{\text{cond}} S_{\text{cond}}$, where the subscripts refer to the valence and conduction band Fermi distributions, respectively. S_{val} and S_{cond} are defined as in (2), but the summation is constrained to states in the valence and conduction band, respectively.
The conservation of $K_I + F_{\text{el}}$ comes from the fact that our GAP potentials have been trained on Mermin free energies and ionic forces computed by application of the Hellmann-Feynman theorem on the internal energy E, as routinely implemented in ab initio codes. We point out that these forces mathematically represent the gradient of the Mermin free energy in presence of a finite electronic temperature, as demonstrated by Wentzcovitch *et al.*[3]. To the best of our knowledge, all previous ab initio calculations performed in order to study non-thermal melting defined energies and forces in the same way, from the Mermin functional.

However, the quantity that is physically conserved in the melting process is the energy, while the entropic terms are not. For this reason, in principle the simulations should be carried out in the NV$(K_I + E)$ ensemble and not in the NV$(K_I + F_{\text{el}})$ one. However, as in the quasi equilibrium picture we consider bands that are not completely filled (i.e. we are not in presence of a closed shell system), we have to introduce fractional electron and hole occupations.

As discussed by Wentzcovitch *et al.*, including fractional occupations implies that E now depends on both the electronic density and the occupations f_i, so that ionic forces computed as $-\nabla_{\mathbf{r}} E$ acquire an additional term describing changes in E due to changes in f_i. This additional term is a correction to usual ionic forces obtained by application of the Hellmann-Feynman theorem on E. Wentzcovitch *et al.* also show that by replacing E with F_{el}, a new contribution $-\nabla_{\mathbf{r}}(TS)$ coming from the electronic entropy appears in $-\nabla_{\mathbf{r}} F_{\text{el}}$ and exactly cancels the term arising from changes in E due to changes in f_i. This implies that Hellmann-Feynman forces computed from E are the true gradient of F_{el} and MD trajectories in the microcanonical ensemble with Hellmann-Feynman forces guarantee the conservation of $K_I + F_{\text{el}}$.

So, switching to the $\mathrm{NV}(K_I + F_{\mathrm{el}})$ ensemble, although practically the most convenient choice, formally conserves a different quantity compared to the physically correct one, i.e. $K_I + E$. The difference lies in the inclusion of the electronic entropy term TS. As a consequence, MD in the $\mathrm{NV}(K_I + F_{\mathrm{el}})$ ensemble provides a trajectory that is different from the physically motivated one, that should be obtained in the $\mathrm{NV}(K_I + E)$ ensemble with the non-Hellmann-Feynman correction to forces. However, if the entropic contribution to the free energy is negligible, as it happens at low temperatures, then the $\mathrm{NV}(K_I + E)$ and the $\mathrm{NV}(K_I + F_{\mathrm{el}})$ ensembles provide equivalent results.
Our goal is then to estimate the error due to running MD in the $\mathrm{NV}(K_I + F_{\mathrm{el}})$ ensemble with Hellman-Feynman forces with respect to the simulation in the $\mathrm{NV}(K_I + E)$ ensemble. One way to do that is to estimate the relative magnitude of the non-Hellmann-Feynman correction to forces, i.e. the relative magnitude of $-\nabla_{\mathbf{r}}(TS)$ with respect to $-\nabla_{\mathbf{r}}F_{\mathrm{el}}$. The bigger the relative contribution arising from $-\nabla_{\mathbf{r}}(TS)$, the bigger we expect the error on the estimation of ionic forces to be, and ultimately the less reliable MD trajectories will be.

We proceed in the following way: we take one random 54-atom diamond crystal structure and we compute F_{el}, E and TS with QE for the structure with the two Fermi level approach assuming 0.2 photoexcited electrons/holes per Si atom with an electronic temperature of $0.01\,\mathrm{Ry} = 1579\,\mathrm{K}$. These values are analogous to those used for QE calculations on photoexcited gapped phases while generating the training set. Then, we displace the first atom of the structure by $\delta l = 0.02\,\text{Å}$ in Cartesian direction x and recompute F_{el}, E and TS. δF_{el}, δE and $\delta(TS)$ are computed as the Mermin free energy, internal energy and entropic term differences between the displaced-atom calculation and the original one. It must then be $\delta F_{\mathrm{el}} = \delta E - \delta(TS)$. A rough estimate of $-\nabla F_{\mathrm{el}}$, $-\nabla E$ and $-\nabla(TS)$ is then given by $-\delta F_{\mathrm{el}}/\delta l$, $-\delta E/\delta l$ and $-\delta(TS)/\delta l$, respectively. We then repeat the calculation 54×3 times by displacing each atom in each Cartesian direction by δl and recomputing δF_{el}, δE and $\delta(TS)$ in each case. Last, we compute the average relative magnitude of $\delta(TS)$ over all applied displacements by adapting Eq. (1):

$$\eta = \sqrt{\frac{\sum_{i=1}^{N_{\mathrm{at}}}\sum_{j=1}^{3}\left|\delta F_{\mathrm{el}_{i;j}} - \delta E_{i;j}\right|^2}{\sum_{i=1}^{N_{\mathrm{at}}}\sum_{j=1}^{3}\left|\delta F_{\mathrm{el}_{i;j}}\right|^2}} = \sqrt{\frac{\sum_{i=1}^{N_{\mathrm{at}}}\sum_{j=1}^{3}\left|\delta(TS)_{i;j}\right|^2}{\sum_{i=1}^{N_{\mathrm{at}}}\sum_{j=1}^{3}\left|\delta F_{\mathrm{el}_{i;j}}\right|^2}}, \tag{3}$$

where the index i runs over all atoms in the structure and j over the Cartesian direction x, y or z in which the displacement δl is applied. We compute η in this way in order to better sample the potential energy surface around the initial structure (since the structure is a distorted crystal that does not have any symmetry) and get a more reliable value. We obtain $\eta \sim 5.7\%$. Increasing δl to $0.04\,\text{Å}$ gives $\eta \sim 5.4\%$, i.e. the result does not depend relevantly on the magnitude of the small displacement. We then repeat the same procedure with a random 64-atom amorphous configuration from our dataset by modeling the electron gas as a single Fermi distribution at a temperature of $0.01\,\mathrm{Ry} = 1579\,\mathrm{K}$. The results are $\eta \sim 4.1\%$ for both $\delta l = 0.02\,\text{Å}$ and $\delta l = 0.04\,\text{Å}$.
These figures must be compared with the relative force errors of our GAP potentials, that are between 13.1% and 16.6% as discussed in Sec. S1. This means that the relative error due to the inaccurate description of ensemble dynamics in our database is below the intrinsic error of GAP potentials, and can thus be safely neglected. We remind that the intrinsic relative error of our GAP potential is analogous to state-of-the-art results, as discussed in Sec. S1. The opposite happens for the ab initio calculations that were performed in previous works by assuming a very high electronic temperature. In fact, the $\delta(TS)$ term has a very strong dependence on the electronic temperature T, while δE has a weaker one. This means that the relative magnitude of $\delta(TS)$ in δF_{el} is relevantly enhanced as T is increased. We prove this by taking the crystalline and amorphous structures considered above and repeating the same procedure, apart from assuming that now electrons obey a single Fermi distribution at a temperature of $0.1\,\mathrm{Ry} = 15790\,\mathrm{K}$. The resulting values of η are 33% for the crystal structure and 29% for the amorphous structure, for both $\delta l = 0.02\,\text{Å}$ and $\delta l = 0.04\,\text{Å}$. These figures are substantially bigger than the claimed force accuracy of previous fitted potentials available in literature (that is for example around 10-15% in [2] and 6-26% in [1]). We believe that previous works that represent the electron-hole plasma as a very hot Fermi distribution suffer from this major issue, i.e. ionic forces computed by their potential have a bigger discrepancy compared to the true energy conserving forces, and the resulting MD trajectories are thus less reliable. On the contrary, the estimated error on our forces is well within the precision of our GAP potentials and is expected to impact MD trajectories only marginally, thanks to the correct treatment of the electron gas. We perform a last test by recomputing η for the crystal structure assuming a double Fermi distribution with 0.2 photoexcited electrons/holes per Si atom and electron and hole temperatures of $0.1\,\mathrm{Ry} = 15790\,\mathrm{K}$. It results $\eta \sim 13.7\%$ for both values of δl employed above, sign that correctly modeling the electron-hole plasma relevantly reduces the non-Hellmann-Feynman force correction even at very high electron and hole temperatures.

Anyway, it is very difficult to estimate the true, practical effect of a 5% or 13% error due to the wrong treatment of the ensemble. An answer to this point might come from the assumption that discrepancies between ML predictions and the ab initio data the ML potential is fitted onto behave in a similar way as discrepancies between the used ensemble and the correct one. If this is the case, a force precision of 4-5% in ML potentials usually corresponds to a force RMSE of around $0.02\,\mathrm{eV/Å}$. This RMSE value is very low and usually implies good convergence of many

properties with respect to the ab initio reference, including very sensitive ones like the phonon dispersion of crystal phases. A force precision of 13% in ML potentials corresponds to force RMSEs of $0.07 - 0.08\,\mathrm{eV/Å}$. This RMSE value usually provides good convergence of properties of disordered phases (like radial and angular distribution functions, structure factors, etc.) and a somehow less satisfactory description of ordered phases. To conclude, we believe that a 4-5% deviation can provide overall better results. A 13% deviation might be more inaccurate especially in the dynamics of the crystal phase, but in some situations could still provide physically reasonable results.

B. Rdf estimation

We estimate the RDF of liquid silicon in the following way. First, we initialize a simulation box containing 512 atoms ($4 \times 4 \times 4$ supercell of the conventional cubic cell) in their ideal lattice sites; we melt the system at $T = 2000\,\mathrm{K}$ for 100 ps, i.e. 10^5 timesteps of 1 fs. Then we change the temperature to the desired value and continue the MD simulation for other 500 ps. We see that the system thermalizes very well to the new temperature in the first 200 ps of the simulation. Thus, we use only the last 300 ps of the simulation to find the average RDF as computed by LAMMPS internal routine and other quantities.

Figure 2 in the main text shows a comparison between our data and AIMD and experimental data by Jakse *et al.*[4]. Jakse *et al.* run AIMD in the local density approximation (LDA) for the exchange correlation functional, while our GAP potential is fitted in the GGA approximation with an ultrasoft PBE pseudopotential, as written in the Methods section in the main text. We believe that the different treatment of the exchange-correlation functional explains the different RDF peak positions in Figure 2.

We now compare our GAP potential with the original GAP-18 paper by Bartók *et al.*[5]. In Figure 7 Bartók *et al.* show RDFs from AIMD and GAP-18 on a 64-atom box at a temperature of 2000 K. For this reason, we run additional MD simulations with LAMMPS in the NPT ensemble at $T = 2000\,\mathrm{K}$ using 64-atom and 512-atom boxes. Fig. S1 shows the comparison between our GAP potential and GAP-18. There is no system size dependence between 64 and 512 atoms and peak positions are correctly described. There is a difference in height of the first peak, that might be due to the different GGA parametrization (PBE in our case, PW91 in the case of Bartók *et al.*) or maybe due to the different pseudizations used.

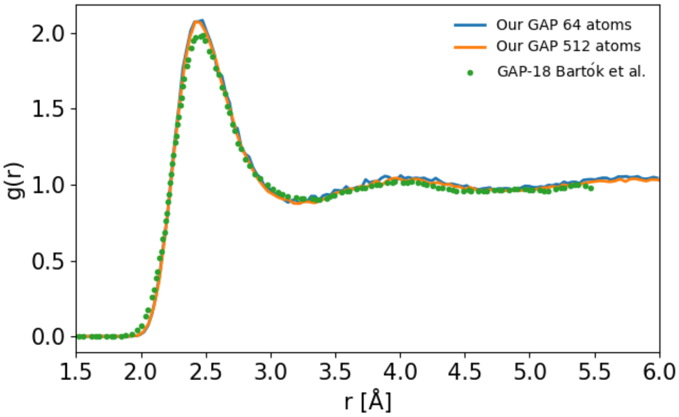

FIG. S1: Comparison between GAP-18 data extracted from Fig. 7 by Bartók *et al.*[5] and 64 and 512-atom simulations with our GAP potential. All calculations are done at 2000 K. Bartók *et al.* use a 64-atom cell. Our simulations are done in the NPT ensemble.

C. Interface simnulations

We estimate the melting point of ground state silicon in the following way:

- We perform a MD simulation of crystalline silicon at zero pressure and at a temperature close to the melting point (1500 K in our case). We employ a 512-atom system and simulate it for 500 ps in the NPT ensemble to correctly relax its volume and atomic positions. The MD timestep is set to 1 fs and we use Nosé-Hoover thermostats and barostats with damping of 0.1 and 1 ps, respectively.

- We perform a MD simulation of a 512-atom liquid system at the same temperature and with the same simulation parameters. The liquid is generated by melting the crystal at 2000 K for 100 ps, then changing temperature to 1500 K and thermalizing it for additional 500 ps.
- The final configurations of the crystal and liquid simulations described above are the starting points of the interface simulation. However, they have two different volumes, but to correctly run an interface simulation we need a perfect overlap between the faces of the two systems. Thus, we rescale the volumes of the two systems to the average between the equilibrium volumes of the crystal and the liquid at 1500 K.
- We then run the proper interface simulation. Since the interface is artificial, atoms around it might be unphysically close to each other, so we run a small NVE equilibration to relax their positions. The NVE simulation is 5 ps long and the excess kinetic energy of atoms close to the interface is removed by rescaling the temperature of the system to the value it had at timestep 0 of the NVE relaxation. After that, we run a NPT simulation of 500 ps with the same parameters described above and look at which phase is prevalent.

We run interface simulations at various temperatures with a spacing of 10 K. We find that the crystal phase is always prevalent up to 1520 K, while the liquid phase prevails from 1530 K on. This method is adapted from the one used by Morris and Song[6].
Our rough estimate of the melting point at zero pressure is very close to the one obtained by Bartók *et al.*[5] through a solid-liquid interface simulation containing 432 atoms, as one can see from Fig. 10 in their work. As stated in the main text, from their work we retained the same training set and the same GGA level of approximation for QE calculations, while we changed the specific pseudopotential, energy and density cutoffs and the k-point mesh density. The replication of their result is a further confirmation of the correctness of our procedure. This leads us to hypothesize that other features of the phase diagram of silicon in that figure could be reproduced similarly as well, like the negative slope of the solid-liquid coexistence line in the $P-T$ diagram. However, such topics are out of the scope of the present work and have not been investigated further.
Estimating the melting temperature with superior precision is outside the scope of this work, as well. Our main interest is to obtain a rough estimate of the melting temperature to further validate our potential. However, our potential can in principle be used to run interface simulations on much bigger systems of tens of thousands of atoms, providing very precise estimates of the melting temperature, due to the very good scaling of GAP potentials. Moreover, an accurate estimation of the melting temperature would also require to correctly treat the electronic temperature in the simulations. In fact, as shown by Geng and Mohn[7], using a fixed electronic temperature in dataset generation (and as well in ab initio MD) can lead to a wrong estimation of the melting temperature.

S3. PHOTOEXCITED SILICON PHASES

Fig. S2 and S3 show parity plots for the energy, forces and stress for two GAP potentials: the ground state, i.e. non photoexcited, one, and the one obtained in the case of 0.2 photoinduced electrons/holes per Si atom. The plots show that our GAP potentials maintain good accuracy throughout the whole regime of fluences investigated.

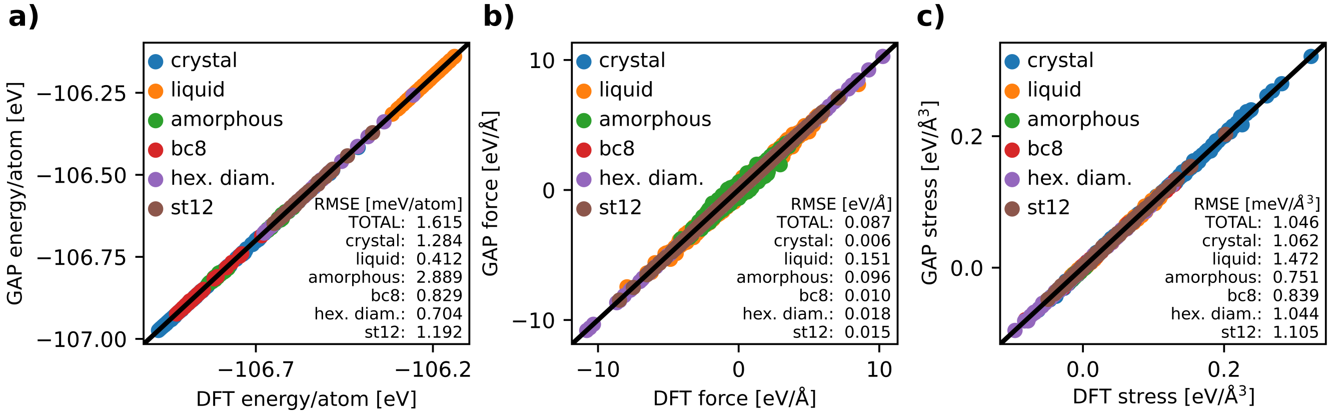

FIG. S2: Parity plots for the ground state, i.e. non photoexcited, potential for (a) energy, (b) forces and (c) stress. The text in the bottom right of the figures is a per-structure report of fit RMSEs, that are close to the usual reference values of 1 meV/atom for the energy and 0.1 eV/Å for the forces.

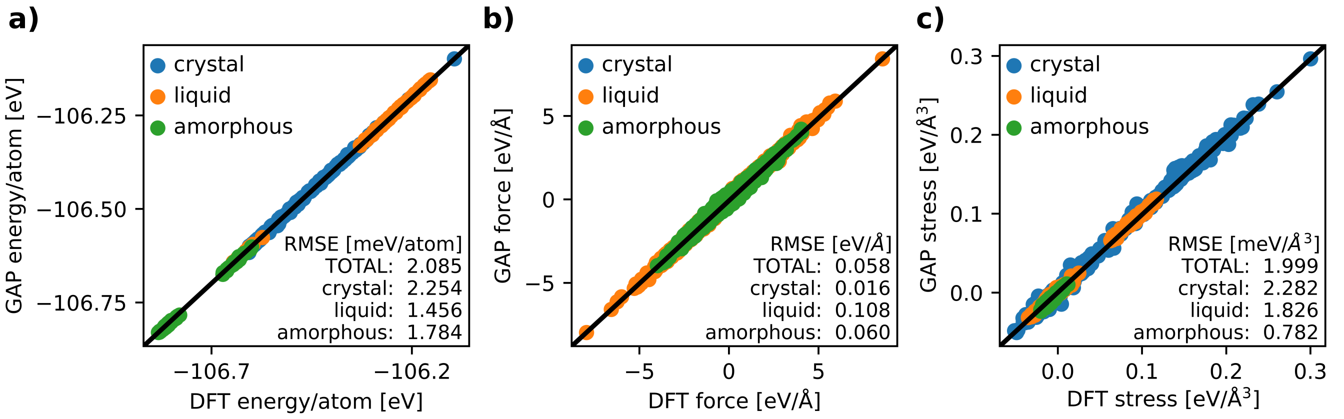

FIG. S3: Parity plots for the potential with 0.2 photoinduced electrons/holes per Si atom for (a) energy, (b) forces and (c) stress. The text in the bottom right of the figures is a per-structure report of fit RMSEs, that are close to the usual reference values of 1 meV/atom for the energy and 0.1 eV/Å for the forces.

S4. ROLE OF THE ELECTRONIC TEMPERATURE AND OF THE TWO FERMI LEVELS IN THE PHONON DISPERSION

As stated in the main text, treating the electron-hole plasma as a single hot Fermi distribution can lead to inaccurate results, and to a wrong attribution of the origin of non-thermal melting. Here, we further substantiate this comment by plotting the phonon dispersion of bulk crystalline silicon computed in 4 different scenarios: by modeling the electrons with one and two Fermi distributions and with two different temperatures for electrons (and holes, where present) of 0.01 Ry and 0.1 Ry. The results are shown in Fig. S4. Panel (a) shows the results for one single Fermi distribution. The low-temperature result (0.01 Ry) is the setup for ground state phonon calculations and is identical to Fig. 1 in the main text. The high-temperature result (0.1 Ry) is similar to phonon calculations performed by Recoules *et al.*[8], Bauerhenne *et al.*[1] and Plettenberg *et al.*[2]. Our results confirm that increasing the electronic temperature smoothens the phonon dispersion, sign that the potential energy surface has less complex features overall. This is proven by the flattening of the optical phonons and the flattening of wiggles at L. In their works, Recoules *et al.* obtain a total collapse of the transverse acoustic (TA) branch in the ΓX and ΓL directions to negative frequencies. This is probably due to the extremely high electronic temperature of 2.15 eV $\sim$ 2500 K used in their calculations, while Bauerhenne *et al.* and Plettenberg *et al.* did the calculation at $18000-19000$ K and our bigger electronic temperature corresponds to 0.1 Ry $\sim$ 1.3 eV $\sim$ 15800 K. It is reasonable to expect that increasing the electronic temperature to the value used by Recoules *et al.*, the TA phonon branch would continue softening almost rigidly until becoming imaginary. However, this is also associated with an unphysical growth of the equilibrium volume of crystalline silicon by some %, contrary to the almost constant value that is observed if photoexcited electrons are treated with the double Fermi level, as discussed in the main text.
Panel (b) shows the results when the electron-hole plasma is accounted for by a double Fermi distribution. The low-temperature result (0.01 Ry) corresponds to the setup for our phonon calculations in Fig. 3 in the main text with 0.2 photoexcited electrons/holes per atom. The high-temperature result (0.1 Ry) is a comparison with high-temperature calculations done by the authors listed above with the single Fermi distribution. In this case, increasing the electronic temperature also smoothens the phonon dispersion and notably recovers instabilities that were present at lower temperatures, confirming that the nesting features of the potential energy surface are smeared out by higher electronic temperatures and disappear. This also implies that optical phonons have a higher frequency at higher electron and hole temperatures. This shows that accurately including the presence of the electron-hole plasma has a relevant impact on the physical interpretation of phonon softening, and ultimately of the onset of non-thermal melting.
We underline that in experiments, increasing fluence at fixed laser frequency corresponds to simultaneously increasing the number of photoexcited electrons and their temperature. This does not represent the situation with 0.2 photoexcited electrons/holes per Si atom and $T_e = 0.1$ Ry in Fig. S4(b). In fact, an increase of the electronic temperature at fixed number of photocarriers can only be obtained by strongly increasing the laser frequency without altering the fluence. This is in principle doable but, in typical optical experiments, the laser frequency can be only tuned in the visible/UV range, which means at most by a factor of 2.

In order to simulate an experimental situation with a higher fluence at fixed frequency, we must at the same time increase the number of photoexcited electrons and the electronic temperature. In this limit, the two Fermi level

approach will correctly lead to unstable phonons and consequently to non-thermal melting, as observed experimentally.

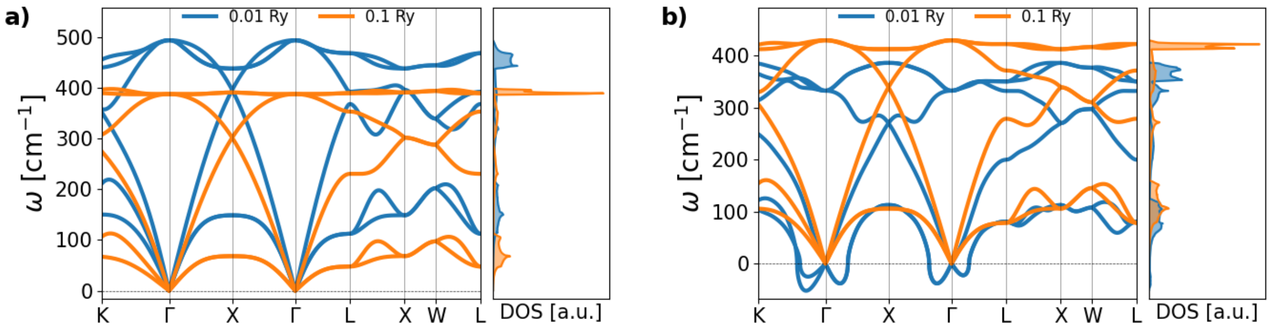

FIG. S4: Phonon dispersion of silicon for various treatments of the electron-hole plasma: (a) phonon dispersion of silicon where the electrons are treated with one single Fermi distribution at a low and a high electronic temperature (0.01 Ry, blue line, and 0.1 Ry, orange line); (b) phonon dispersion of silicon where the electrons are treated with two Fermi distributions at low and high electron and hole temperatures (0.01 Ry, blue line, and 0.1 Ry, orange line). In both plots, increasing the temperature leads to a smoothening of the phonon dispersion, however photoinduced instabilities in (b) are recovered.

S5. ROLE OF THE SYSTEM SIZE AND SCALING OF GAP POTENTIALS

Here we briefly expand the discussion on the dependence of MD simulations on the system size. In the main text we already showed that the RMSD in the first hundreds of fs of a MD simulation of non-thermal melting is almost indistinguishable for 512-atom MD cells and bigger ones, while 64-atom cells provide quite different result (Fig. 5(d) in the main text). Here we bring the analysis further by plotting the Debye-Waller factor, RMSD and ionic temperature as a function of system size for cells containing 512, 13824 and 32768 atoms and for longer times. Agreement between our data at 512 atoms and bigger cells is impressive in all plots and shows that 512-atom systems already provide a good description of the thermodynamic limit and of the dynamics of non-thermal melting, as shown in Fig. S5(d-f). For times longer than 200 fs, we find that the smallest simulation box (64 atoms) undergoes spurious oscillations in RMSD, DW factor and $T_l(t)$ (Fig. S5(a-c)). These oscillations are quickly suppressed for larger cell simulations.
The performance of the GAP potential in these simulations is reported in Table S1 and confirms its almost linear scaling up to cells with tens of thousands of atoms.

	512 atoms	13824 atoms	32768 atoms
Number of CPU cores	16	96	96
Speed [timestep/s]	0.510	0.134	0.055
Speed per CPU core [timestep/s/CPU]	0.0319	0.0014	0.0006
Speed per CPU core per atom [timestep×atom/s/CPU]	16.30	18.49	18.87

TABLE S1: Performance of GAP potentials for various system sizes (512, 13824 and 32768 atoms). The last line shows that the scaling of GAP potentials up to system sizes of 32768 atoms is almost linear.

S6. ROLE OF THE ELECTRONIC TEMPERATURE IN MD SIMULATIONS OF NON-THERMAL MELTING

This section expands the discussion in the main text about the role of the electronic temperature T_e used in the fitting of the GAP potentials. In Fig. S6 we show the comparison between simulations with 0.2 photoinduced electrons/holes per Si atom and two different electronic temperatures, namely 0.01 Ry and 0.025 Ry. Fig. S6(a-c) is identical to Fig. 9 in the main text and reports MD data at $T_l(0) = 300\,\mathrm{K}$, together with experimental data from Ref. [10]. Fig. S6(d-f) shows a similar plot for MD simulations with $T_l(0) = 10\,\mathrm{K}$, for which no experimental data are available. The discrepancy between the timescales for non-thermal melting with two different electronic temperatures increases as $T_l(0)$ is lowered.

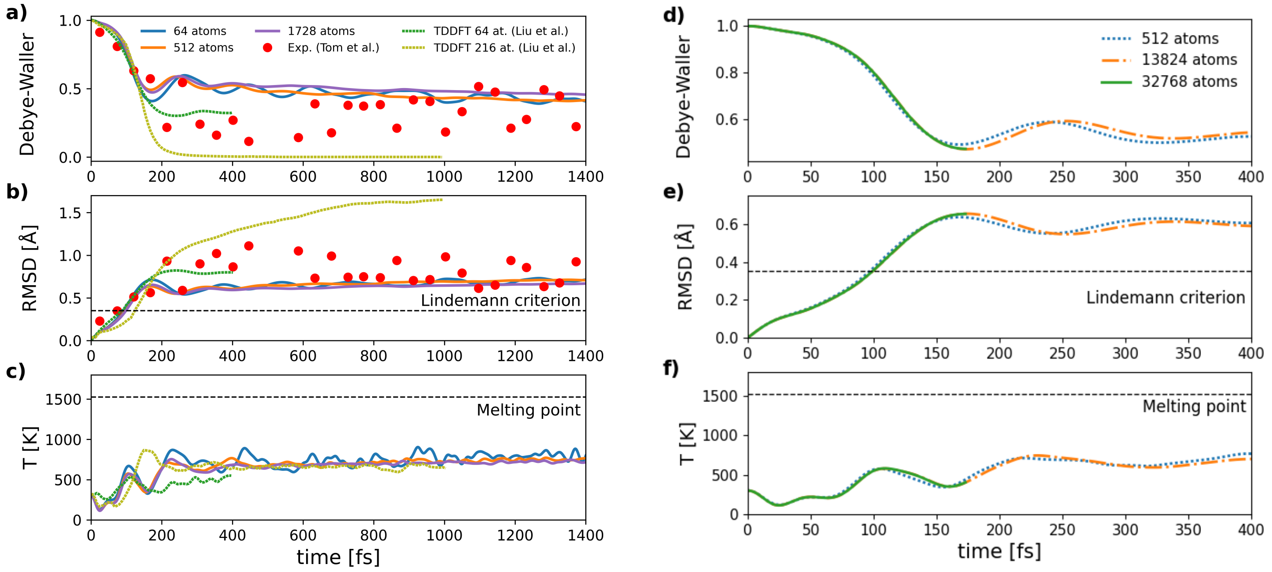

FIG. S5: More results for MD simulations of non-thermal melting with the GAP potential at 0.2 photoinduced electrons/holes per Si atom and $T_l(0) = 300\,\mathrm{K}$. (a-c) Replotting of Fig. 5 in the main text with additional lines for 64 atoms (blue line) and 512 atoms (orange line) to show the system-size dependence of our results. The panels show the time evolution of (a) the Debye-Waller factor in the (200) direction, (b) the root mean square displacement (RMSD) and (c) the lattice temperature during the simulations. The dashed lines show the Lindemann criterion for silicon (0.35 Å) and the theoretical melting temperature of the GAP GS potential ($1520 - 1530\,\mathrm{K}$). After 200 fs, results for 64-atom systems start to show unphysical oscillations, that are quickly suppressed for bigger boxes. Data are also compared with results from real-time TDDFT by Liu *et al.*[9] (green lines) and experiments by Tom *et al.*[10] (red points). (d-f) Focus on the system size dependence of (d) the Debye-Waller factor in the (200) direction, (e) the root mean square displacement (RMSD), (f) the lattice temperature for boxes up to 32768 atoms in the first 400 fs of the simulation.

S7. TWO-TEMPERATURE MODELS AND TIME EVOLUTION OF THE ELECTRONIC TEMPERATURE

Previous works studying ultrafast phenomena in silicon and other materials by using extremely large electronic temperatures also implemented the two-temperature model (TTM) scheme to describe the time evolution of the electronic temperature due to electron-phonon interactions [2, 11–13]. In those works, explicitly including the time evolution of the electronic temperature is strictly necessary due to its extremely large values at the beginning of MD simulations. On the contrary, in our approach the role of the electronic temperature is less central since the non-thermal effect is described by the presence of photoexcited electrons, which remain constrained in the conduction band as long as the diamond phase is present. As a consequence, electrons and holes in the photoexcited diamond phase remain relatively cold ($\approx$ 1000-2000 K), as discussed in Sec. 2.1 in the main text. The electronic system then loses free energy when going to the disordered phases due to the recombination of electrons and holes following gap closure, assuming that all the heat exchange between lattice and electrons is encoded in the structural relaxation following the laser-induced modification of the Mermin free energy and the consequent structural destabilization. This effect is included in our machine learning potential, so we believe that it can adequately describe the first ps after photoexcitation. In doing so, we do not include incoherent electron-phonon scattering channels that also contribute to heat exchange between electrons and phonons. We believe that this effect, while important and worth studying in future works, would not qualitatively change the physical mechanism underlying non-thermal melting described here.

S8. CONVERGENCE OF THE PHONON DISPERSIONS

Figure S7 shows convergence results for the phonon dispersion of crystalline silicon with 0.2 photoexcited electrons/holes per Si atom (same setup as in Fig. 3 in the main). We show ab initio results from QE for 5 different q-point grids with the constrained density functional perturbation theory method. We show GAP results for 4 dif-

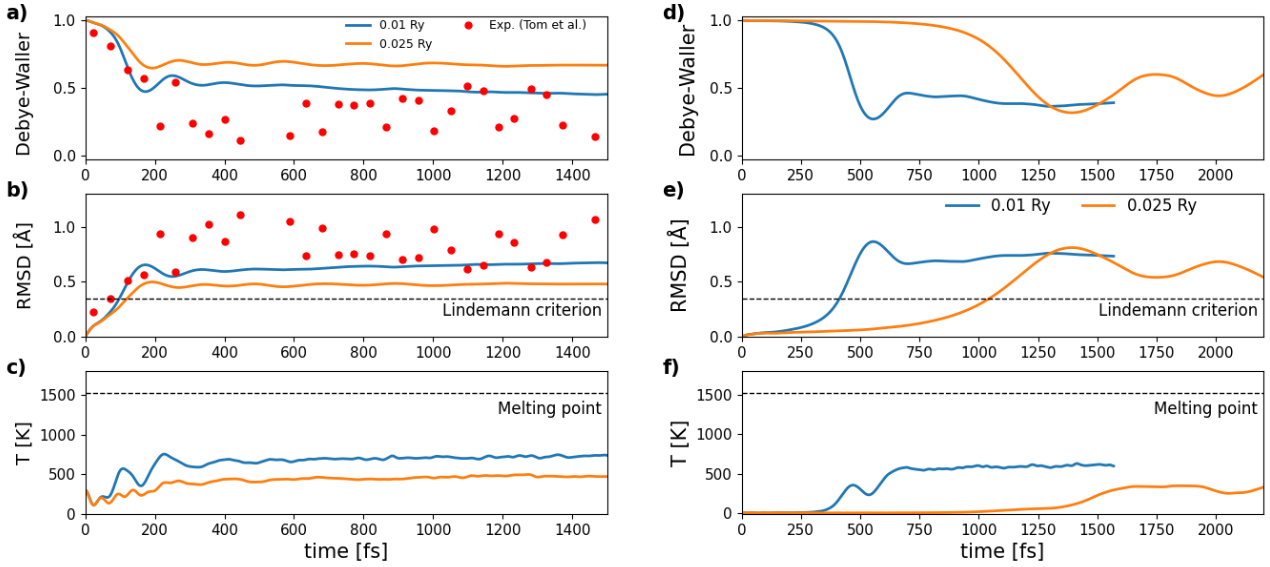

FIG. S6: MD simulations of non-thermal melting with the GAP potential at 0.2 photoinduced electrons/holes per Si atom with two different electronic temperatures (0.01 Ry, blue lines, and 0.025 Ry, orange lines). (a-c) replotting of Fig. 9 in the main text: time evolution of (a) the Debye-Waller factor in the (200) direction, (b) the root mean square displacement (RMSD) and (c) the lattice temperature. MD results refer to NV($K_I + F_{\mathrm{el}}$) simulations with 1728 atoms and $T_l(0) = 300\,\mathrm{K}$. Experimental results by Tom *et al.*[10] are also reported (red dots) for a $610-\mathrm{nm}$ laser. Experimental RMSD points are obtained from the Debye-Waller factor applying the inverse of equation (2) in the main text. The dashed lines show the Lindemann criterion for silicon (0.35 Å) and the theoretical melting temperature of the GAP GS potential ($1520-1530\,\mathrm{K}$). (d-f) same plot for MD simulations with a lower initial lattice temperature equal to $T_l(0) = 10\,\mathrm{K}$.

ferent supercell sizes with the finite displacement method. GAP phonons are exactly identical for all supercells from $5\times5\times5$ or bigger, since the cell becomes larger than twice the cutoff of the GAP potential. This is a limitation of strictly local machine learning potentials, however the overall good agreement between converged QE and GAP results (Fig. 3(a) in the main) suggests that long-range contributions to the GAP dynamical matrix are not so relevant.

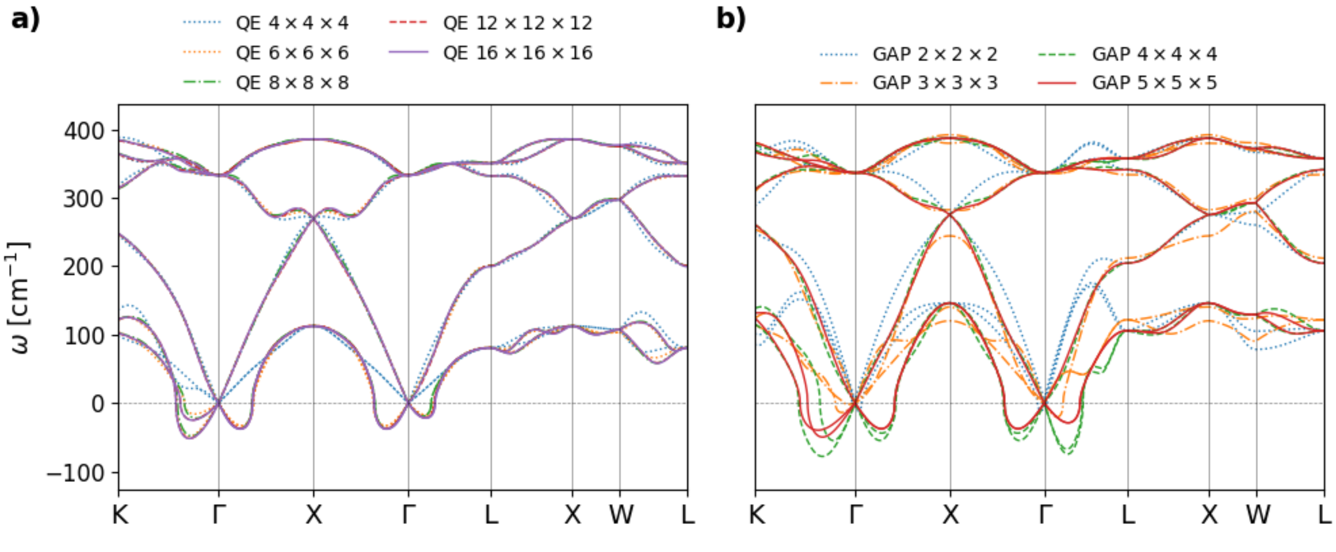

FIG. S7: Convergence tests of the phonon dispersion of silicon: (a) convergence for the ab initio phonon dispersion with q-point mesh; (b) convergence of the finite displacement GAP phonon dispersion with supercell size.

S9. HEXAGONAL DIAMOND, BC8 AND ST12 CONFIGURATIONS

In this Section, we explain the reasons why we removed all hexagonal diamond, bc8 and st12 configurations from the training set at fluences larger than 2.5%.

Laser photoexcitation generates a population of free carriers in the conduction band of silicon, so the insulating behavior of the crystal phase is suppressed and a metallic character emerges. At the same time, the stability of each crystal polymorph of silicon changes as a function of photoexcitation in a different and phase-specific way. This increases a lot the complexity of the photoexcited potential energy surface that the GAP potential has to learn and the quality of the fit is strongly degraded.

However, we perform some tests in non-photoexcited silicon and we see that excluding the hexagonal diamond, bc8 and st12 structures from the training set does not generally change the properties of the diamond and liquid phases of silicon. We then assume that the same would hold for the photoexcited potentials, i.e. a good description of non-thermal melting can be attained without including the hexagonal diamond, bc8 and st12 configurations in the photoexcited dataset. In this way, we describe a potential energy surface that is less complex but still contains all the relevant features for non-thermal melting, and the GAP potential can learn it accurately. Moreover, the removed structures only provide 2.2% of the atomic environments of our training set.

Fig. S8(a) compares the cohesive energy per atom for all crystal structures between the non-photoexcited dataset and the one at 2.5% fluence. Fig. S8(b) shows a similar comparison for the Cartesian components of atomic forces. A simple and clear reason for fit quality degradation does not emerge clearly from this plot: energies and forces are a bit different but no anomalous behavior is present. Cohesive energies align in different regions of the plane because the main contribution to changes in cohesive energy upon photoexcitation comes from bandgap energy, and the average bandgap value is again phase specific.

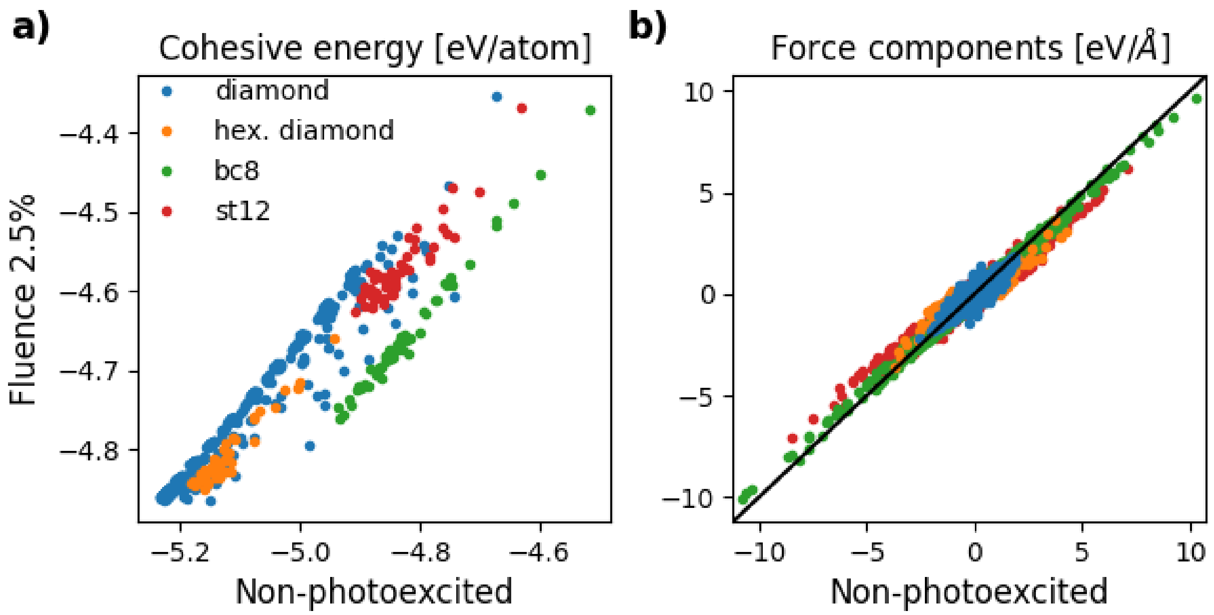

FIG. S8: Comparison between the crystalline configurations (diamond, hexagonal diamond, bc8 and st12) in our non-photoexcited dataset and in the one at 0.2 photoexcited electrons/holes per Si atom: (a) comparison of cohesive energies per atom; (b) comparison of the Cartesian components of atomic forces.

We tried to retain hexagonal diamond, bc8 and st12 structures in the photoexcited datasets by varying various hyperparameters, especially the regularization parameters σ_{energy}, σ_{forces} and σ_{virial}. We would have accepted the obtained GAP potentials if they had satified these two constraints:

- give a very accurate description of the phonon dispersion of the diamond phase, that is fundamental to accurately describe non-thermal melting. We find that converging the phonon dispersion gets harder as fluence is increased, requiring a force RMSE on the diamond phase smaller than 0.2 eV/Å for the case of 0.2 photoexcited electrons/holes per Si atom;

- provide overall good RMSEs over all the training set.

Unfortunately, no attempt was successful. The Figures below show three example parity plots for three GAP potentials fitted with the database at 0.2 electrons/holes per Si atom including the hexagonal diamond, bc8 and st12 configurations.
The first fit (Figure S9) is done by increasing the default force regularization parameter to $\sigma_{\text{forces}} = 0.1\,\text{eV/Å}$ as in the original fitting of GAP-18. The overall quality of the fit is not bad, but the force RMSE on the diamond phase is $0.043\,\text{eV/Å}$, too high to provide a reasonable phonon dispersion. We also show the phonon dispersion provided by this potential, that describes in a totally wrong manner the instabilities in the acoustic branch. The second fit (Figure

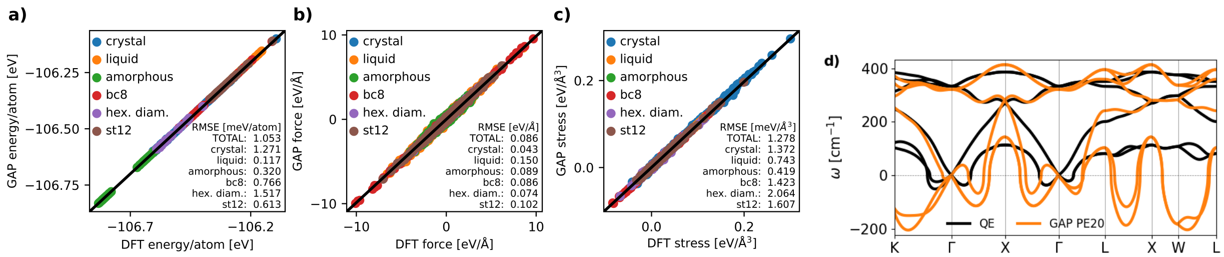

FIG. S9: Parity plot for a GAP potential with $\sigma_{\text{forces}} = 0.1\,\text{eV/Å}$ for all crystalline structures (diamond, hexagonal diamond, bc8 and st12) for (a) energy, (b) forces and (c) stress; (d) phonon dispersion of the diamond crystal provided by the potential.

S10) is done with $\sigma_{\text{forces}} = 0.01\,\text{eV/Å}$ for all crystal structures. We then wondered if using different regularization

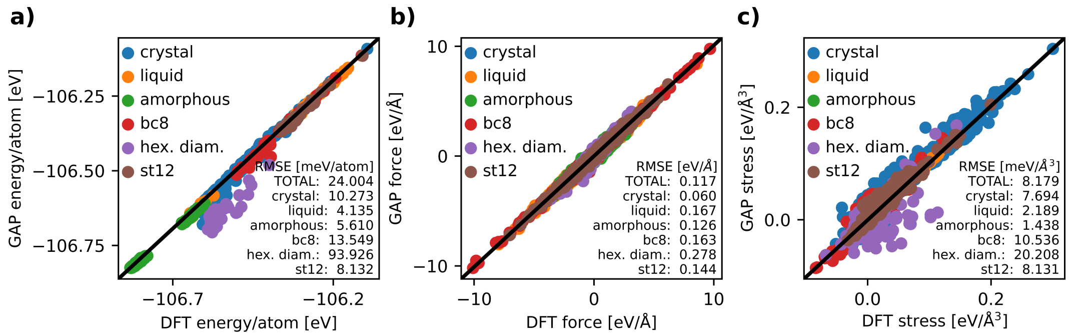

FIG. S10: Parity plot for a GAP potential with $\sigma_{\text{forces}} = 0.01\,\text{eV/Å}$ for all crystalline structures (diamond, hexagonal diamond, bc8 and st12) for (a) energy, (b) forces and (c) stress.

parameters for the various phases could improve the fit. This is not the case, as shown in Figure S11 for a GAP potential with $\sigma_{\text{forces}} = 0.01\,\text{eV/Å}$ for the diamond phase only and $\sigma_{\text{forces}} = 0.1\,\text{eV/Å}$ for the hexagonal diamond, bc8 and st12 phases. (σ_{energy} and σ_{virial} are also doubled compared to the values used for the diamond phase).

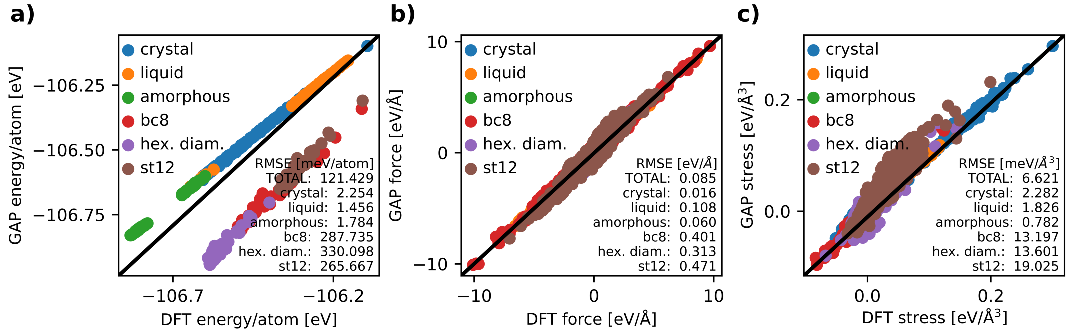

FIG. S11: Parity plot for a GAP potential with $\sigma_{\text{forces}} = 0.01\,\text{eV/Å}$ for the diamond phase and $\sigma_{\text{forces}} = 0.1\,\text{eV/Å}$ for the hexagonal diamond, bc8 and st12 phases for (a) energy, (b) forces and (c) stress.

S10. LIMITATIONS AND APPLICABILITY OF THE PRESENT WORK TO OTHER SYSTEMS

Our work is based on the hypothesis of a clear timescale separation between electron thermalization processes (on the fs timescale) and electron-phonon interactions, that typically activate about $150-200\,\mathrm{fs}$ after excitation. An explicit treatment of the electrons is necessary whenever electron-phonon interactions play a relevant role in the dynamics of the system. So, if a photoinduced melting process occurs on a timescale between few fs and $150-200\,\mathrm{fs}$, an explicit treatment of the electrons in the study of the melting process is not necessary. We believe that this is also true for insulators with strong (but well-behaved) electron-phonon coupling, since the timescale at which the electron-phonon coupling becomes relevant does not greatly depend on its strength.
One could then notice that MD simulations were run for longer times up to $\sim 1\,\mathrm{ps}$. So, contributions due to incoherent electron-phonon scattering processes could become relevant in these simulations, further enhancing ionic motion and decreasing the scattering intensity accordingly, although it is very difficult to evaluate the magnitude of this effect.

Our work presents a simple model for a quartic potential where a lattice instability has developed. Thus, in principle, it is meaningful to apply it whenever the structural transition is driven by an imaginary phonon. In the present case, the instability develops at finite momentum near Γ almost isotropically, and a one-dimensional order parameter is enough to capture the behavior of the system near the phase transition. Conversely, other systems may need a multidimensional model to qualitatively reproduce the real behavior, as the instability could be driven by several soft phonons. This case is, however, a simple generalization of the 1D model to higher dimensions. More complicated would be the case where the whole phonon branch goes imaginary at the same time and an order-disorder transition would be observed. In this case our model would not apply.
Thus, as a general idea, it is reasonable to assume that the same model would work in the description of other tetravalent materials in similar photoexcitation conditions, provided that the same behavior of localized (in the Brillouin zone) phonon instabilities is observed, and in any other case where the observed phonon instability is localized.

[1] B. Bauerhenne, V. P. Lipp, T. Zier, E. S. Zijlstra, and M. E. Garcia, Phys. Rev. Lett. **124**, 085501 (2020).
[2] P. Plettenberg, B. Bauerhenne, and M. E. Garcia, Communications Materials **4**, 63 (2023).
[3] R. M. Wentzcovitch, J. L. Martins, and P. B. Allen, Phys. Rev. B **45**, 11372 (1992).
[4] N. Jakse, L. Hennet, D. L. Price, S. Krishnan, T. Key, E. Artacho, B. Glorieux, A. Pasturel, and M.-L. Saboungi, Applied Physics Letters **83**, 4734 (2003), https://pubs.aip.org/aip/apl/article-pdf/83/23/4734/18584430/4734_1_online.pdf.
[5] A. P. Bartók, J. Kermode, N. Bernstein, and G. Csányi, Phys. Rev. X **8**, 041048 (2018).
[6] J. R. Morris and X. Song, The Journal of Chemical Physics **116**, 9352 (2002), https://pubs.aip.org/aip/jcp/article-pdf/116/21/9352/19109997/9352_1_online.pdf.
[7] M. Geng and C. E. Mohn, Phys. Rev. B **108**, 134110 (2023).
[8] V. Recoules, J. Clérouin, G. Zérah, P. M. Anglade, and S. Mazevet, Phys. Rev. Lett. **96**, 055503 (2006).
[9] W.-H. Liu, J.-W. Luo, S.-S. Li, and L.-W. Wang, Science Advances **8**, eabn4430 (2022), https://www.science.org/doi/pdf/10.1126/sciadv.abn4430.
[10] H. W. K. Tom, G. D. Aumiller, and C. H. Brito-Cruz, Phys. Rev. Lett. **60**, 1438 (1988).
[11] E. S. Zijlstra, A. Kalitsov, T. Zier, and M. E. Garcia, Phys. Rev. X **3**, 011005 (2013).
[12] R. Darkins, P.-W. Ma, S. T. Murphy, and D. M. Duffy, Phys. Rev. B **98**, 024304 (2018).
[13] Q. Zeng, B. Chen, S. Zhang, D. Kang, H. Wang, X. Yu, and J. Dai, "Full-scale ab initio simulations of laser-driven atomistic dynamics," (2023).
